# SARS-CoV-2 reshapes m^6^A methylation in long noncoding RNAs of human lung cells

**DOI:** 10.1093/narmme/ugaf034

**Published:** 2025-09-30

**Authors:** Cristina M Peter, Caio O Cyrino, Nilmar S Moretti, Fernando Antoneli, Marcelo R S Briones

**Affiliations:** Center for Medical Bioinformatics, Escola Paulista de Medicina, Federal University of São Paulo (UNIFESP), São Paulo 04039-032, SP, Brazil; Center for Medical Bioinformatics, Escola Paulista de Medicina, Federal University of São Paulo (UNIFESP), São Paulo 04039-032, SP, Brazil; Departamento de Microbiologia, Imunologia e Parasitologia, Escola Paulista de Medicina, Federal University of São Paulo (UNIFESP), São Paulo 04023-062, SP, Brazil; Center for Medical Bioinformatics, Escola Paulista de Medicina, Federal University of São Paulo (UNIFESP), São Paulo 04039-032, SP, Brazil; Center for Medical Bioinformatics, Escola Paulista de Medicina, Federal University of São Paulo (UNIFESP), São Paulo 04039-032, SP, Brazil

## Abstract

*N*^6^-Methyladenosine (m^6^A) is a key base modification that regulates RNA stability and translation during viral infection. While m^6^A methylation of host mRNAs has been studied in SARS-CoV-2-infected cells, its role in long noncoding RNAs (lncRNAs) is unknown. Here, we analyzed direct RNA sequencing (dRNA-seq) data from infected human lung cells (Calu-3) using a machine learning m^6^A detection framework. We observed a global increase in m^6^A levels across 10 antiviral response–associated lncRNAs, with UCA1, GAS5, and NORAD—regulators of interferon (IFN) signaling—showing the most pronounced changes. This might, in part, explain the attenuated IFN expression observed in infected cells. We identified methylated DRACH motifs in predicted lncRNA duplex-forming regions, which may favor Hoogsteen base-pairing, which destabilize secondary structures and target interaction sites. These results provide new perspectives on how SARS-CoV-2 could impact lncRNAs to modulate host immunity and viral persistence through m^6^A-dependent mechanisms.

## Graphical Abstract

**Graphical Abstract:**
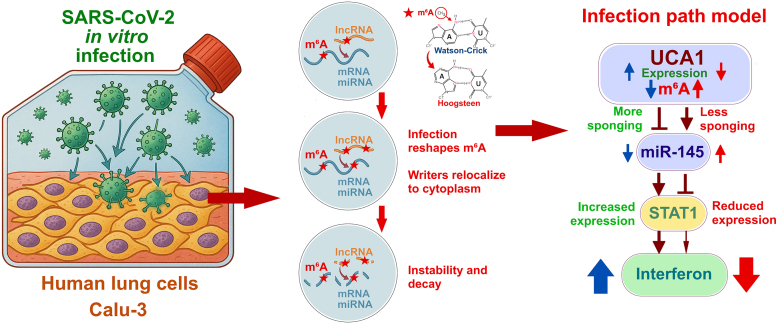

## Introduction

Severe acute respiratory syndrome coronavirus 2 (SARS-CoV-2), the etiological agent of COVID-19 [1], is a positive-sense single-stranded RNA virus that replicates in the cytoplasm of infected cells [2, 3]. In addition to viral RNAs, SARS-CoV-2-infected cells harbor diverse endogenous transcripts, including transfer RNAs (tRNAs), ribosomal RNAs (rRNAs), messenger RNAs (mRNAs), long noncoding RNAs (lncRNAs), and microRNAs (miRNAs), all of which may be affected by viral infection [4, 5]. SARS-CoV-2 disrupts host gene expression and post-transcriptional regulation [6], including the methylation landscape of host mRNAs via *N*^6^-methyladenosine (m^6^A), the most prevalent and functionally significant RNA modification in eukaryotic cells [7].

Co-transcriptional m^6^A methylation occurs in the third position (adenosine) in DRACH motifs (D = A/G/U; R = A/G; A; C; H = A/C/U) [8] by a methyltransferase complex composed of METTL3, METTL14, and WTAP (writers) [9]. This base modification is reversible, with FTO and ALKBH5 acting as demethylases (erasers) [10, 11], and is interpreted by m^6^A proteins such as YTHDFs and IGF2BPs (readers), which modulate RNA stability, splicing, translation, and decay [12, 13]. SARS-CoV-2 hijacks this machinery to enhance viral RNA stability and escape from immune detection [5, 14]. Notably, METTL3 interacts directly with the viral replication complex, facilitating m^6^A modification of viral RNAs and contributing to replication efficiency and interferon (IFN) evasion [15].

LncRNAs are at least 500 nucleotides long, do not contain coding regions, and are emerging as key regulators of cellular homeostasis and immune responses [16]. They function as molecular scaffolds, sponges, decoys, and guides for chromatin modifiers, RNA binding proteins (RBPs), and miRNAs [17–19]. In the context of SARS-CoV-2 infection [20], several lncRNAs are differentially expressed and implicated in cytokine modulation, IFN signaling, and viral genome processing [6, 21]. Yet, despite their importance, the epitranscriptomic regulation of lncRNAs during infection remains largely unexplored [22].

In particular, the extent to which m^6^A methylation shapes lncRNA function during SARS-CoV-2 infection is unknown. Given that lncRNAs often act through RNA–RNA or RNA–protein interactions, modifications that alter RNA structure could have significant regulatory consequences. Whether m^6^A methylation modulates such interactions—especially in immune-related lncRNAs—is a critical open question.

Here, we examine m^6^A methylation dynamics in lncRNAs from SARS-CoV-2-infected Calu-3 cells, a human lung epithelial cell line that robustly supports infection and mirrors many features of *in vivo* airway epithelium [23]. Using direct RNA sequencing (dRNA-seq) data and a machine learning–based detection framework [24], we map high-confidence m^6^A sites across a curated set of immune-regulatory lncRNAs. We identify SARS-CoV-2-induced m^6^A remodeling in lncRNAs enriched at predicted RNA–RNA duplex regions and propose a novel hypothesis, where m^6^A promotes transient Hoogsteen-like pairing [25] in hairpins and base-pairing interfaces, potentially modulating lncRNA interactions and immune signaling.

## Materials and methods

### Direct RNA sequence data

RNA sequences (dRNA-seq reads) from SARS-CoV-2-infected and uninfected Calu-3 cells were obtained from [23]. Calu-3 cells were infected with SARS-CoV-2/Australia/VIC01/2020, NCBI: MT007544.1. These cells are widely used as an *in vitro* model for studying respiratory viral infections, particularly coronaviruses (SARS-CoV, SARS-CoV-2, and MERS-CoV) and influenza viruses, due to their high expression of angiotensin-converting enzyme 2 (ACE2), the primary receptor for SARS-CoV-2 entry [26, 27]. Calu-3 cells were infected with SARS-CoV-2 for 48 h, followed by total RNA extraction and dRNA-seq. Reads from control and infected Calu-3 cells were obtained from SRA BioProject PRJNA675370, SRA Accession #SRR13089335 of SARS-CoV-2-infected Calu-3 RNA reads and #SRR13089334-uninfected Calu-3 RNA reads [23]. The data are from three separate infections of the same cell line performed at the same time and are, therefore, technical replicates rather than biological replicates. While replicates were sequenced separately for cDNA-seq, they were pooled prior to dRNA-seq due to technical limitations in multiplexing with Oxford Nanopore Technologies (ONT) sequencing kits. Whole blood transcriptome data of COVID-19 patients and respective controls were obtained from PRJNA718349, samples GSE171110 and GSE157103 [28].

### Contig assembly by mapping to references

For the identification of lncRNAs of interest in response to SARS-CoV-2, we initially conducted a search in the NCBI (National Center for Biotechnology Information) database using specific terms related to viral infection and lncRNA expression. After selecting the relevant, we downloaded the records in FASTA format, ensuring the preservation of structure and integrity. After downloading it in FASTA format, we used Minimap2 to map the dRNA-seq reads to the lncRNA reference sequences [29, 30]. This mapping allowed us to identify the presence and potential variations in lncRNA expression in response to viral infection, providing insights into their possible role in regulating the cellular response to the virus. The Minimap2 mapping generated BAM files for posterior analyses. The expression levels were estimated as fold change (FC) from the number of reads mapped to each lncRNA in infected and uninfected cells using the formula:


(1) \begin{eqnarray*}{\mathrm{F}}{{\mathrm{C}}_{{\mathrm{lncRNA}}}} = \left( {{N_{{\mathrm{ri}}}}/{T_{{\mathrm{ri}}}}} \right)/\left( {{N_{{\mathrm{ru}}}}/{T_{{\mathrm{ru}}}}} \right)
\end{eqnarray*}

where *N*_ri _= number of mapped reads of each lncRNA in infected cells, *T*_ri _= total mapped reads in infected cells (1 055 956), *N*_ru _= number of mapped reads of each lncRNA in uninfected cells, and *T*_ru _= total reads in infected cells (940 040).

### Methylation analysis

From the BAM files and the fast5 files from the Nanopore sequencing the “index” and “eventalign” modules from Nanopolish (v0.14.1.) were utilized for the signal segmentation step, commonly referred to as “squiggling.” The segmented raw signals obtained in this step were stored in the eventalign output file and subsequently pre-processed using the m^6^A net-dataprep module. The detection of m^6^A modifications within DRACH motifs was performed via the m^6^A net-run_inference algorithm, both of which are implemented in m6anet (v2.1) [24]. The complete workflow, including the custom scripts used for data processing and analysis, is available online: https://zenodo.org/records/16883738.

### Analysis of DRACH motifs

For detailed inspection of methylated DRACH motifs—checking for information bias—the consensus sequences were obtained from the variation calling step. This procedure was performed with Longshot (v.0.4.1), with default settings for long reads, and establishing a minimum threshold of read depth for ×30 coverage to accept the SNV occurrence [31]. The resulting VCF files obtained in this step were used to generate consensus by the “bcftools consensus” - BCFTools (v.1.15) [32]. All methylated DRACH motifs were subsequently extracted and flanked by five nucleotides on each side to enable sequence alignment and stacking. This procedure was implemented using a custom computational pipeline developed in Python (version 3.13.2) [33].

To minimize the occurrence of m^6^A sites that may represent false positives in Calu-3 cell samples (the presence of methylated m^6^A sites with a reduced probability of predictions as a function of many transcripts/reads) and that could add noise to the analysis, the inspection of the DRACH motifs was performed using m^6^A sites with ≥0.6 probability of modification threshold as calculated by m6anet [24]. To reduce the occurrence of false negatives in SARS-CoV-2 with fewer reads, and a smaller genome size, the methylation probability threshold was set at ≥0.6. Because some DRACH motifs (including five nucleotides at each end) can be located at the coordinate ends of cellular transcripts and viral RNAs, resulting in truncated sequences, the removal of these sequences was necessary. The analysis of nucleotide biases within DRACH motifs was conducted using the ggseqlogo package (v.0.2) implemented in R (version 4.4.2) [34].

### Statistical tests of differential methylation

To compare the distribution of methylated sites per transcript in sequencing reads from control and infected cells, we performed the Wilcoxon–Mann–Whitney (WMW) test, as implemented in R version 4.4.2 within the base R package [35, 36]. This nonparametric test is widely used to assess differences between two independent groups when their distributions do not meet the assumptions of normality. Under the null hypothesis (H₀), the distributions of both groups are assumed to be identical, whereas the alternative hypothesis (H₁) posits that the distributions differ significantly, specifically by detecting a difference in their medians.

To apply the WMW test to our data, we first gathered all predictions generated by m^6^A net and stored them in tables, which were subsequently imported into R as data frames and labeled as “uninfected” and “infected.” We then applied a probability threshold (modification probability ≥ 0.6) to filter out sites with low confidence in m^6^A modification. The primary variable chosen to compare the methylation distribution between the two groups was the number of reads per site (*n* reads). Since the number of reads per site is not standardized by default, data normalization was required. For this, the number of reads per site in each group was divided by the total number of reads in that group using the equation below. After normalization, the data underwent a negative logarithm transformation (−log) to enhance visualization consistency across samples:


(2) \begin{eqnarray*}{n_{\frac{{\textit{reads}{\mathrm{\;}}\textit{normalized}}}{{\textit{site}}}}} = \frac{{{n_{\frac{{\textit{reads}}}{{\textit{site}}}}}}}{{\sum {n_{\frac{{\textit{reads}}}{{\textit{site}}}}}}}
\end{eqnarray*}

To visualize the distributional differences between groups, we generated violin plots using the ggplot2 package (version 3.5.1) in R [37]. Violin plots provide comprehensive graphical representation of data distribution by combining a box plot with a kernel density estimate, enabling the visualization of both summary statistics and the underlying probability density function.

### Prediction of RNA interactions and integration with m^6^A methylation analysis

Interactions between lncRNAs and coding mRNAs were predicted using the LncRRIsearch platform (http://rtools.cbrc.jp/LncRRIsearch), which allows for the systematic identification of RNA–RNA interactions based on sequence complementarity and structural accessibility [38]. This methodology considers thermodynamic parameters and RNA secondary structure to predict potential base-pairing regions between transcripts. The lncRNAs GAS5, NORAD, and UCA1, in their reference isoforms, were used as input for the interaction predictions. Target mRNAs were selected based on their relevance to antiviral response, IFN signaling, and inflammatory regulation based on experimental evidence related to SARS-CoV-2 infection [39].

For each predicted interaction, nucleotide positions corresponding to the interaction region between the lncRNA and mRNA were extracted. These data were then integrated with epitranscriptomic profiles generated through dRNA-seq (Oxford Nanopore Technology) and analysis with m6anet for the identification of m^6^A sites. The relationship between methylation and RNA–RNA interactions, we looked at overlaps between predicted binding regions and canonical DRACH motifs, which define the consensus sequence recognized by the m^6^A methyltransferase complex. Only interactions coinciding with high confidence m^6^A sites (as predicted by m6anet) were retained for downstream analysis. Methylation events were categorized according to their genomic context: m^6^A in the lncRNA, when the modification occurred within the lncRNA region involved in the interaction, m^6^A in the target gene, when located within the interacting region of the target mRNA and m^6^A in both, when m^6^A sites were detected in both interacting partners. The lncRNA–SARS-CoV-2 interactions were predicted using intaRNA [40] and confirmed by RNAhybrid program [41]. RNAfold was used for secondary structure analysis [42].

## Results

### Mapping dRNA-seq reads to lncRNAs

A curated set of 100 lncRNAs was assembled from published data based on their reported involvement in SARS-CoV-2 infection. Inclusion criteria encompassed associations with (i) inflammation, (ii) apoptosis, (iii) biomarker potential, (iv) innate immune response, and (v) adaptive immune response (Supplementary Table S1). dRNA-seq reads were aligned to lncRNA reference sequences using Minimap2. From the initial set, 34 lncRNAs with higher read coverage were selected for downstream analyses, as greater read depth improves the reliability of methylation site detection (Supplementary Table S2). BAM files corresponding to these 34 lncRNAs were analyzed for m^6^A modifications using a multiple instance learning framework (m6anet). Among them, 10 lncRNAs exhibited strong evidence of m^6^A methylation—defined as prediction probabilities exceeding 60%—predominantly in infected cells. These 10 lncRNAs were selected for detailed downstream analyses, as summarized in Table 1.

**Table 1.** Set of immune-related lncRNAs expressed in uninfected Calu-3 cells (U) and SARS-CoV-2-infected Calu-3 cells (I)

LncRNA	Gene ID	Description	Target	Regulation	Mapped reads (U)	Mapped reads (I)	Reference
BISPR	ENSG00000282851	BST2 IFN-stimulated positive regulator	BST2 (tetherin)	*Cis*	13 594	15 728	[20]
GAS5	ENSG00000234741	Growth arrest specific 5	TLR4, IL-6, TNF-α, SIRT1​miR-155	*Trans*	2247	2655	[43]
LINC00278	ENSG00000231535	Long intergenic nonprotein coding RNA 278	IL-6 TNF-α​	*Trans*	9734	11 510	[44]
LINC00511	ENSG00000227036	Long intergenic nonprotein coding RNA 511	STAT3 NF-κB	*Trans*	14 194	16 440	[45]
MIR155HG	ENSG00000234883	MIR155 host gene	NF-κB, IRF7 IFNAR1	*Trans*	1348	1804	[20]
NORAD	ENSG00000260032	Noncoding RNA activated by DNA damage	PUM1, NF-κB (indirect), p53	*Trans*	3587	4467	[46]
THRIL	ENSG00000280634	HNRNPL-related immunoregulatory lncRNA	TNF-α, IL-6, IL-1β	*Trans*	3787	4323	[47]
UCA1	ENSG00000214049	Urothelial cancer-associated 1	IL-6, TNF-α, NF-κB (via miRNAs)	*Trans*	137	129	[48]
CASC2	ENSG00000177640	Cancer susceptibility 2	NF-κB, IL-6, TNF-α	*Trans*	12 819	15 102	[49]
LINC01619	ENSG00000257242	Long intergenic nonprotein coding RNA 1619	FOXO1 miR-27a-3p	*Trans*	14 926	17 445	[44]

These lncRNAs are a subset of curated a lncRNA set of 100 lncRNAs based on their relevance in immune response and inflammation regulation in SARS-CoV-2 infection. A total of 940 040 reads from uninfected cells and 1 055 956 reads from infected cells were analyzed.

The dRNA-seq reads of infected and uninfected samples were mapped to the 10 selected lncRNA reference sequences. A total of 940 040 dRNA-seq reads from uninfected cells (U) and 1 055 956 dRNA-seq reads from infected cells (I) were analyzed. The expression profiles of selected lncRNAs associated with the NF-κB, IFN-stimulated gene (ISG), *FOXO1*, and STAT3 pathways in uninfected and infected cells are summarized in Table 1. Most of the lncRNAs were predicted to act via *trans*-regulatory mechanisms, targeting genes related to inflammation, immune signaling, and antiviral defense. BISPR was the only *cis*-acting lncRNA, located adjacent to the ISG *BST2* (tetherin).

### Expression of lncRNAs in SARS-CoV-2-infected human lung cells

**Figure 1.**
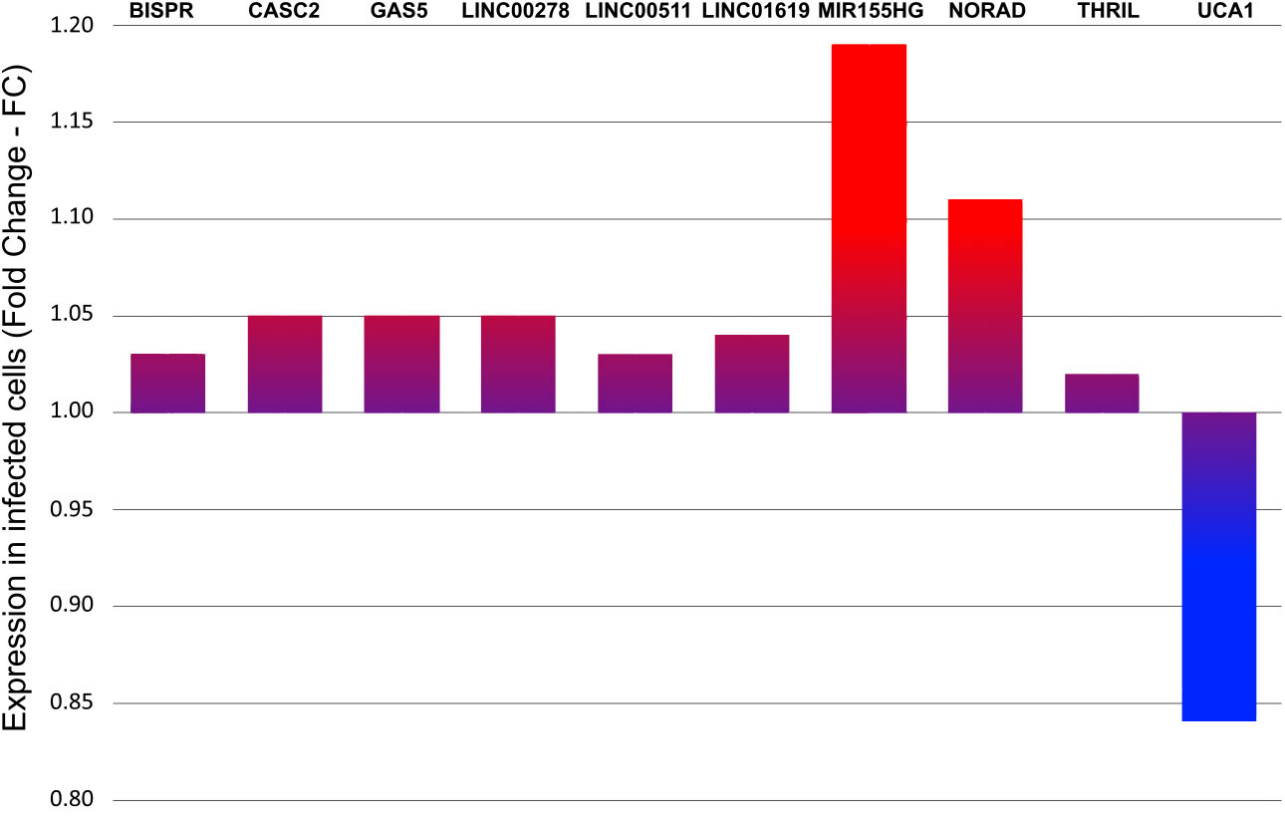
Expression of immune-associated lncRNAs in SARS-CoV-2-infected human cells (Calu-3). Bars represent the normalized FC of 10 lncRNAs, associated with immune pathways, in SARS-CoV-2-infected cells as compared to uninfected cells. Values were derived from reference-based alignment of dRNA-seq data (BioProject PRJNA675370) and quantified using mapped read counts. Red indicates upregulation FC > 1.10, purple indicates 1.05 > FC > 1.00, and blue indicates downregulated (FC < 1.00) in infected cells.

The expression profiles of the 10 lncRNAs detailed in Table 1 were estimated from dRNA-seq data by normalization of reads mapped to the lncRNA reference sequences (Fig. 1). Most of the selected lncRNAs displayed modest upregulation upon infection, with fold changes ranging from ~1.03 to 1.06. Among upregulated lncRNAs, GAS5, LINC00511, MIR155HG, THRIL, and NORAD stood out due to their roles in inflammatory signaling. GAS5 and NORAD, both regulators of NF-κB, exhibited increased expression in infected samples, supporting their role in host immune defense. Notably, MIR155HG exhibited the highest increase in expression (FC ≈ 1.19), consistent with its known role in inflammatory and antiviral responses. NORAD also showed a marked upregulation (FC ≈ 1.11). In contrast, UCA1 was the only lncRNA to show a substantial decrease in expression, with a fold change <0.90.

### Global changes of m^6^A patterns in SARS-CoV-2 infection

**Figure 2.**
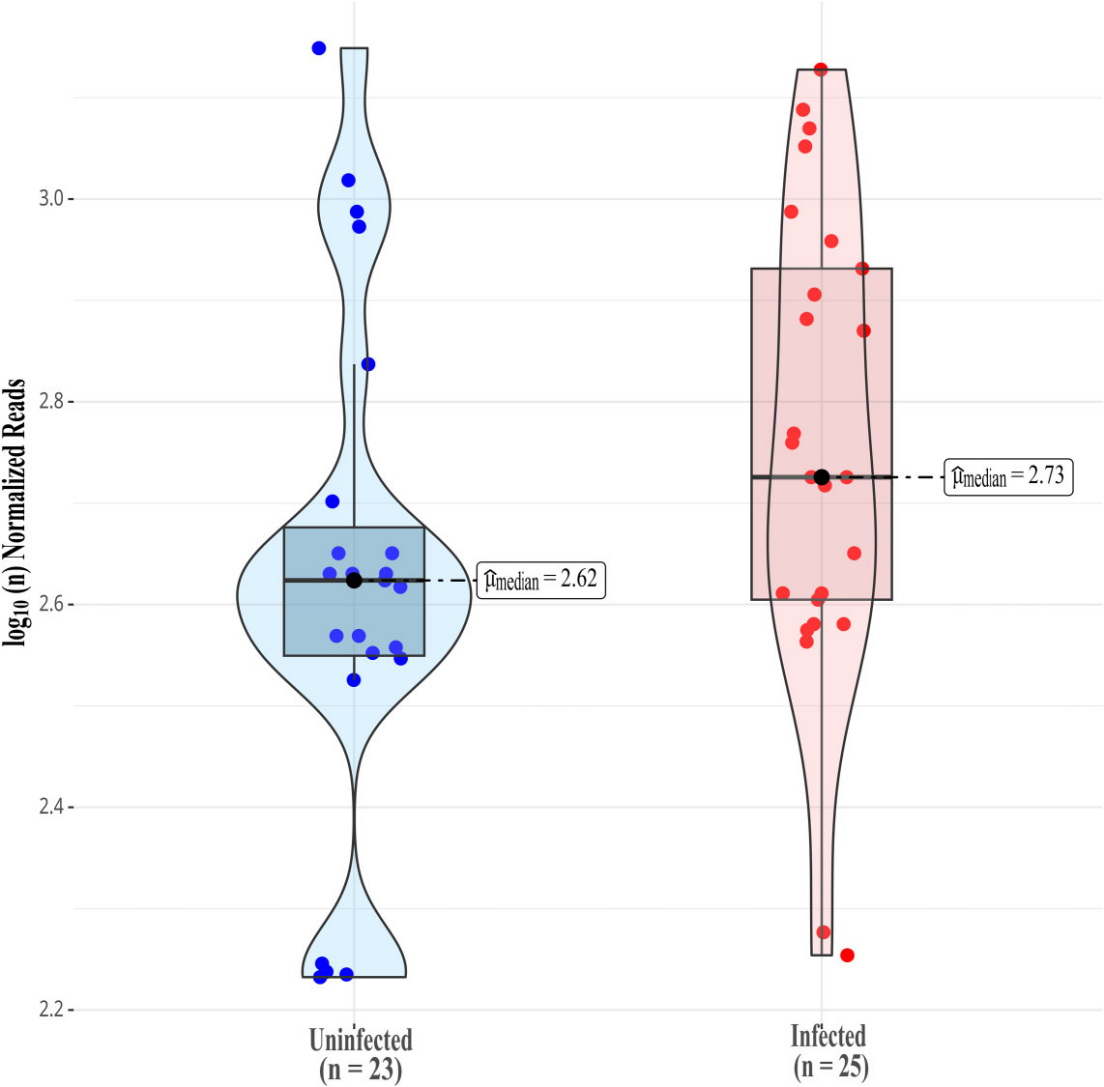
Global distribution of m^6^A sites in 10 lncRNAs of SARS-CoV-2-infected and uninfected Calu-3 cells. The shaded areas of violin plots represent data distributions, and the horizontal bars indicate the medians. Methylation is estimated by multiple instance learning framework. Effect size and *P*-values were calculated using the Wilcoxon–Mann–Whitney test. The *x*-axis represents the sample groups, and the *y*-axis denotes *S* = −log (standardized reads), which is the base-10 logarithm of the proportion of methylated sites by the number of normalized reads in each dataset. The number of methylated sites is *n* = 23 in uninfected cells and *n* = 25 in infected cells with *W*_Mann–Whitney _= 189.50, *P *= 0.04, *ȓ*_biserial_ = −0.34, CI_95%_ = [−0.60, −0.02], *n*_obs _= 48.

To assess whether the observed differences in m^6^A methylation between uninfected and infected samples were statistically significant, we performed a comparative analysis using the normalized methylation reads across all transcripts. This analysis shows the distribution of differentially methylated lncRNAs in Calu-3 cells (Fig. 2). Each violin plot reflects the density of methylation values for uninfected and SARS-CoV-2-infected samples, with median values indicated by horizontal bars. The statistical significance of m^6^A differences between uninfected and SARS-CoV-2-infected Calu-3 cells, was assessed by the Wilcoxon–Mann–Whitney test (nonparametric) to compare normalized methylation signals across all detected sites in 10 selected lncRNAs. The metric used, *S* = −log (standardized reads), represents the base-10 logarithm of the proportion of methylated sites normalized by read count.

The violin plots reveal the distribution of m^6^A values, where each point represents an individual methylated site, and the shaded areas reflect the density of the data (Fig. 2). The infected group (*n* = 25) exhibited a slightly higher median methylation level (2.73) compared to the uninfected group (2.62), suggesting an overall increase in m^6^A modification upon infection. This difference is statistically significant (W = 189.5, *P *= 0.04), with a rank biserial effect size of −0.34, indicating a moderate shift in distribution. The 95% confidence interval (CI = [−0.60, −0.02]) further supports the reliability of this difference. The metric used, *S* = −log_10_ (standardized reads), captures the proportion of methylated sites relative to read-depth in each condition, allowing for normalization across samples.

### Mapping m^6^A modifications in lncRNAs

The regulatory potential of these lncRNAs, beyond their transcriptional changes, was assessed by their m^6^A epitranscriptome profiles. Using the same set of lncRNAs identified in expression analysis, we identified m^6^A-modified sites with methylation probabilities ≥60% (Table 2). m^6^A sites were detected in the 10 selected lncRNAs, including transcript coordinates, DRACH motifs, read counts, and methylation probabilities, revealing transcript-specific modification patterns (Table 2). Especially, several lncRNAs that exhibited transcriptional regulation, such as GAS5, THRIL, MIR155HG, and UCA1, also displayed consistent or increased m^6^A methylation signals following infection. The references used in these analyses were gene sequences so to include all exons of each lncRNA and avoid specificities of splicing variants expressed in different cell types and conditions.

**Table 2.** Distribution of m^6^A sites (*P *≥ 60%) in 10 lncRNA genomic sequences in Calu-3-uninfected cells (U) and SARS-CoV-2-infected cells (I)

LncRNA	m^6^A position	Coverage	Probability	*K*-mer
BISPR (U)	296*	163	60%	UGACC
BISPR (I)	ND	ND	ND	ND
**CASC2 (U)**	**12 758**	**164**	**78%**	**AGACU**
**CASC2 (I)**	**12 758**	**157**	**79%**	**AGACU**
GAS5 (U)	3550	20	83%	AGACA
	3979	63	82%	UGACA
	4062	76	80%	UGACC
GAS5 (I)	3542**	29	81%	GGACA
	3550	31	73%	AGACA
	3979	37	78%	UGACA
	4062	49	68%	UGACC
LINC00278 (U)	ND	ND	ND	ND
LINC00278 (I)	11 652**	48	62%	GAACU
LINC00511 (U)	3163*	84	63%	AGACU
LINC00511 (I)	ND	ND	ND	ND
LINC01619 (U)	7046	67	86%	AGACU
	7144*	165	61%	AGACA
LINC01619 (I)	7046	74	91%	AGACU
**MIR155HG (U)**	**9143**	**76**	**98%**	**AGACA**
**MIR155HG (I)**	**9143**	**54**	**96%**	**AGACA**
NORAD (U)	2483*	27	89%	AAACU
	2799	30	66%	GGACU
NORAD (I)	2192**	21	63%	AGACU
	2661**	23	64%	GGACA
	2799	25	83%	GGACU
**THRIL (U)**	**615**	**160**	**95%**	**UGACU**
**THRIL (I)**	**615**	**149**	**95%**	**UGACU**
UCA1 (U)	2367*	29	91%	GGACC
	5738	56	91%	AGACA
	5922	66	93%	GGACC
	5971*	79	70%	AGACC
	6098	68	77%	GGACA
	6158	66	77%	GGACA
	6197	66	91%	GGACA
	6285	80	61%	GAACU
	6293	78	90%	GAACU
	6390	63	76%	GGACA
	6683	41	86%	GAACU
UCA1 (I)	5638**	53	86%	GGACC
	5738	53	91%	AGACA
	5922	70	96%	GGACC
	6098	69	86%	GGACA
	6158	75	80%	GGACA
	6197	69	92%	GGACA
	6285	77	69%	GAACU
	6293	74	78%	GAACU
	6390	63	90%	GGACA
	6583**	24	75%	AGACU
	6683	33	72%	GAACU
	6917**	35	62%	UGACU
	6934**	38	84%	UGACU

m^6^A sites were identified using multiple instance learning as implemented in m6anet software. Not Detected (ND). Higher m^6^A methylation in infected cells (**) and uninfected cells (*). Bold fonts indicate lncRNAs with invariant m^6^A methylation. Numbering of m6A positions of the gene sequence refers to hg38 human genome assembly. Coverage is the number of reads that support the position. Probability is calculated by m6anet. *K*-mer is the specific DRACH motif where the m^6^A is located.

Several lncRNAs exhibited distinct changes in their m^6^A methylation profiles in infected cells, including both the loss and gain of methylation sites (Table 2). BISPR, which showed upregulation at the transcriptional level, has a m^6^A methylated site (*P *= 60%) in uninfected cells, but no methylation sites were detected post-infection. Similarly, LINC00511 exhibited a methylated site with 63% probability in uninfected cells, whereas no detectable m^6^A signal was observed following infection. However, LINC00278, with no detectable methylation in uninfected cells, acquires a m^6^A-modified site (62%) upon infection.

Several lncRNAs exhibited invariant m^6^A modification patterns in uninfected vs infected cells comparison. THRIL displayed an invariant m^6^A site (with probability 95%) at the same position in both uninfected and infected cells, with a slight decrease in read coverage (Table 2). This suggests a robust and consistent epitranscriptome signature, potentially associated with maintenance of its role in tumor necrosis factor alpha (TNFα) regulation of inflammatory response. Similarly, CASC2 retained a methylation site at the same position in both conditions (78% and 79%) (Table 2). MIR155HG also maintained a highly methylated site (98% in uninfected cells and 96% in infected cells). Other lncRNAs showed m^6^A sites exclusively in infected cells, suggesting infection-induced epitranscriptome remodeling. NORAD exhibited two m^6^A-modified sites in uninfected cells, and three in infected cells, including newly detected positions with methylation probabilities ≥60% (Table 2).

Several lncRNAs exhibited shared m^6^A-modified sites in both uninfected and infected cells, however with variations in methylation probability and/or read coverage, suggesting subtle post-transcriptional regulation rather than a complete gain or loss of modification. GAS5 retained multiple methylation sites in both conditions, although methylation probabilities decreased moderately in infected cells (e.g. from 83% to 73%, and from 82% to 78%), along with an overall decline in read counts (Table 2). NORAD, in addition to gaining new methylation sites, preserved at least one shared site (position 2799), with a methylation increase from 66% to 83% post-infection. Similarly, UCA1 displayed a mixed pattern, with numerous shared sites showing slight increases in methylation probabilities in infected cells (Table 2).

Individual methylation analysis of lncRNA transcripts of the most m^6^A-modified lncRNAs (Table 2) was performed with UCA1 (ENST00000397381.4) (Supplementary Table S3), NORAD (ENST00000565493.1) (Supplementary Table S4), and GAS5 (ENST00000430245.1) (Supplementary Table S5). The transcript analysis confirms the higher methylation of UCA1 (2299 nt) with 48 DRACH motifs where 11 are methylated in infected cells and 12 in uninfected cells with probabilities ≥60% (*P *≥ 60%) (Supplementary Table S3). NORAD (5339 nt) also shows 48 DRACH motifs where three are methylated in infected cells and one in uninfected cells (*P* ≥ 60%) (Supplementary Table S4). GAS5 (723 nt) has 12 DRACH motifs where only one is methylated with probability between 50% and 60% in infected and uninfected cells (Supplementary Table S5). The difference in GAS5 methylation when gene and transcript sequences are compared is due to methylated positions in introns which are identified in Table 2 but that are not present in GAS5 transcript GAS5-027 that has predicted interaction regions with STAT2.

Given NEAT1 established role in viral infection response, we analyzed its main transcript (ENST00000501122.2, NEAT1-001, 22 743 nt) (Supplementary Table S6). NEAT1 is upregulated in dRNA-seq data (FC = 1.1028) and has only one m^6^A methylated position at 16 649 with probability 51.8% in infected cells and 77.3% in uninfected cells. All other DRACH motifs have methylation probabilities below 30%. NEAT1 is predicted to form RNA–RNA interactions with STAT1 and STAT2 (free energy ≤ –16 kcal/mol), which are key effectors in the interferon response pathway. The m^6^A methylated sites are not located in the target interaction regions.

### Changes in nucleotide preferences in DRACH motifs

**Figure 3.**
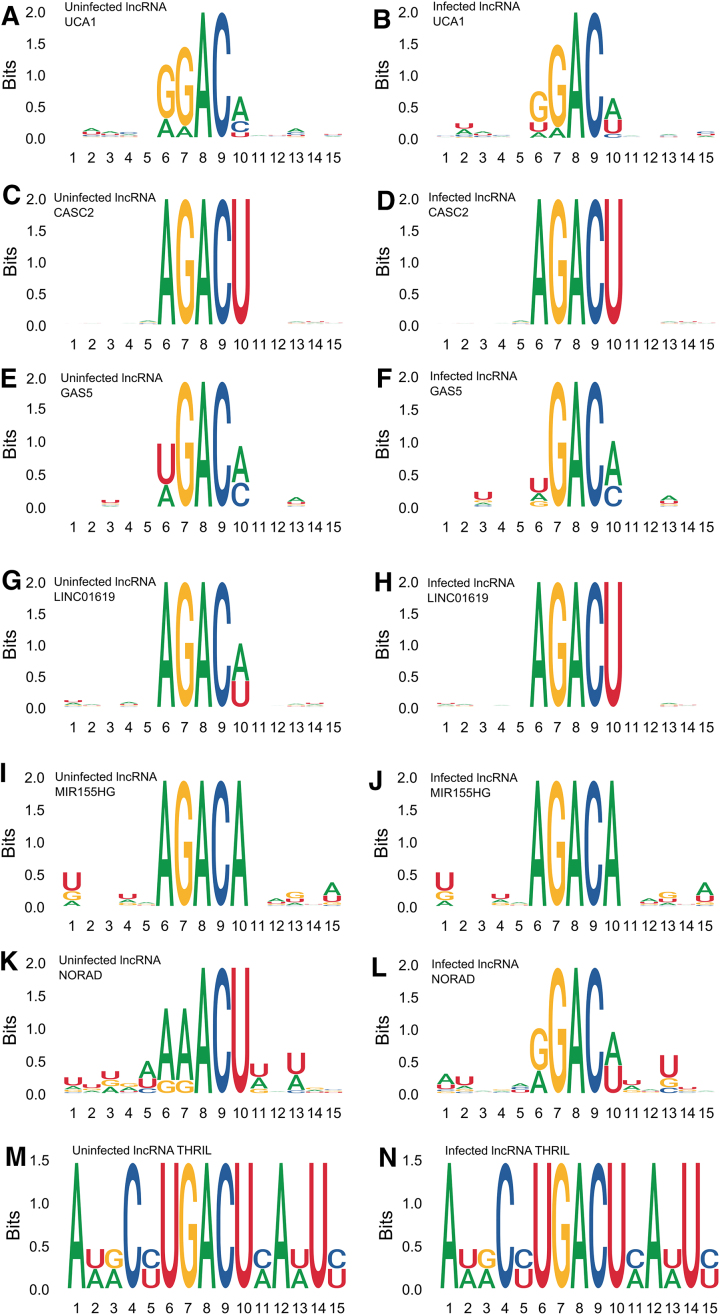
Nucleotide bias in lncRNA methylated DRACH motifs in SARS-CoV-2-infected cells. DRACH sequences containing predicted m^6^A sites (plus five nucleotides for each end) were aligned and stacked together to provide an overview of the informational content of methylated regions. Motif profiles in epitranscriptomes were obtained from Calu-3-uninfected cells (**A, C, E, G, I, K**, and **M**), and from samples of infected Calu-3 cells (**B, D, F, H, J, L**, and **N**). The ordinates indicate the score in bits as it deviates from the null hypothesis (higher scores indicate stronger biases). Results are shown for (A and B) UCA1, (C and D) CASC2, (E and F) GAS5, (G and H) LINC01619, (I and J) MIR155HG, (K and L) NORAD, and (M and N) THRIL.

We investigated whether the sequence context of m^6^A-modified sites also varied in response to SARS-CoV-2 infection. Sequence logo analysis of methylated DRACH motifs shows alterations in nucleotide preferences in several positions (Fig. 3). The analysis was performed by extracting each DRACH motif along with five flanking nucleotides on either side, providing a comprehensive view of the informational content and nucleotide bias surrounding methylated regions. Sequence profiles were generated separately for uninfected Calu-3 cells (Fig. 3A, C, E, G, I, K, and M) and SARS-CoV-2-infected cells (Fig. 3B, D, F, H, J, L, and N). It is important to note that only seven lncRNAs are represented, as three of the 10 selected transcripts did not exhibit detectable m^6^A modifications in either condition. The *y*-axis indicates the information content in bits, where higher values reflect stronger sequence conservation and greater deviation from randomness.

The core DRACH motif—particularly the GAC triplet—was highly conserved under both uninfected and infected conditions, although subtle variations emerged in flanking nucleotides, as summarized in Table 4. In several transcripts, such as GAS5 (Fig. 3E and F), LINC00278 (Fig. 3G and H), and LINC01619 (Fig. 3I and J), we observed an increased frequency of adenine (A) and uracil (U) residues surrounding the DRACH motif in infected cells, suggesting a possible shift in sequence preference for m^6^A deposition during infection. Additionally, in lncRNAs like MIR155HG (Fig. 3K and L) and CASC2 (Fig. 3C and D), the information content (in bits) around the GAC core appeared slightly higher in infected cells, indicating stronger sequence conservation and potentially increased specificity of the m^6^A methylation machinery during SARS-CoV-2 infection. In contrast, BISPR (Fig. 3A and B) maintained a highly similar sequence profile across both conditions, suggesting structural stability of its methylation motif. Finally, lncRNA THRIL revealed a conserved core DRACH motif (Fig. 3M and N) in uninfected and infected cells, indicating canonical recognition by the methyltransferase complex. Notably, subtle variations in flanking nucleotide composition were observed upon infection, potentially reflecting context-specific adjustments in methylation targeting during viral stress.

### Positions of DRACH motifs and methylated m^6^A sites in lncRNAs

**Figure 4.**
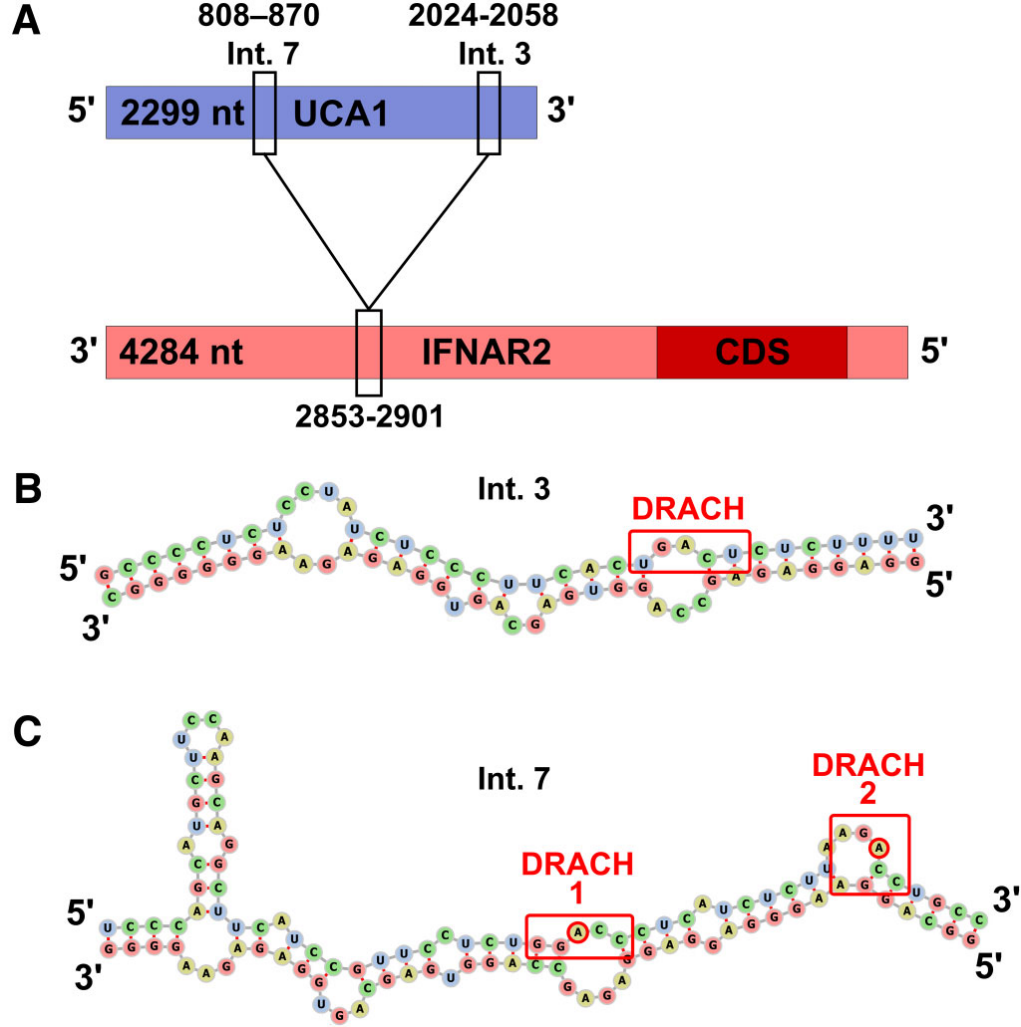
DRACH motifs in lncRNA UCA1 target interaction regions. (**A**) Map of the UCA1–IFNAR2 interaction regions 3 and 7. (**B**) The DRACH motif in interaction region 3 (Int. 3, free energy = −17.82 kcal/mol) with unmethylated A, also unpaired. (**C**) DRACH motifs 1 and 2 in interaction region 7 (Int. 7, free energy = −16.66 kcal/mol) contain methylated A bases, but they don’t pair with the target mRNA. UCA1-001 is the largest UCA1 splicing variant (ENST00000397381.4). IFNAR2 is the interferon-α/β-receptor β-chain mRNA splicing variant IFNAR2-001 (ENST00000404220.3).

**Figure 5.**
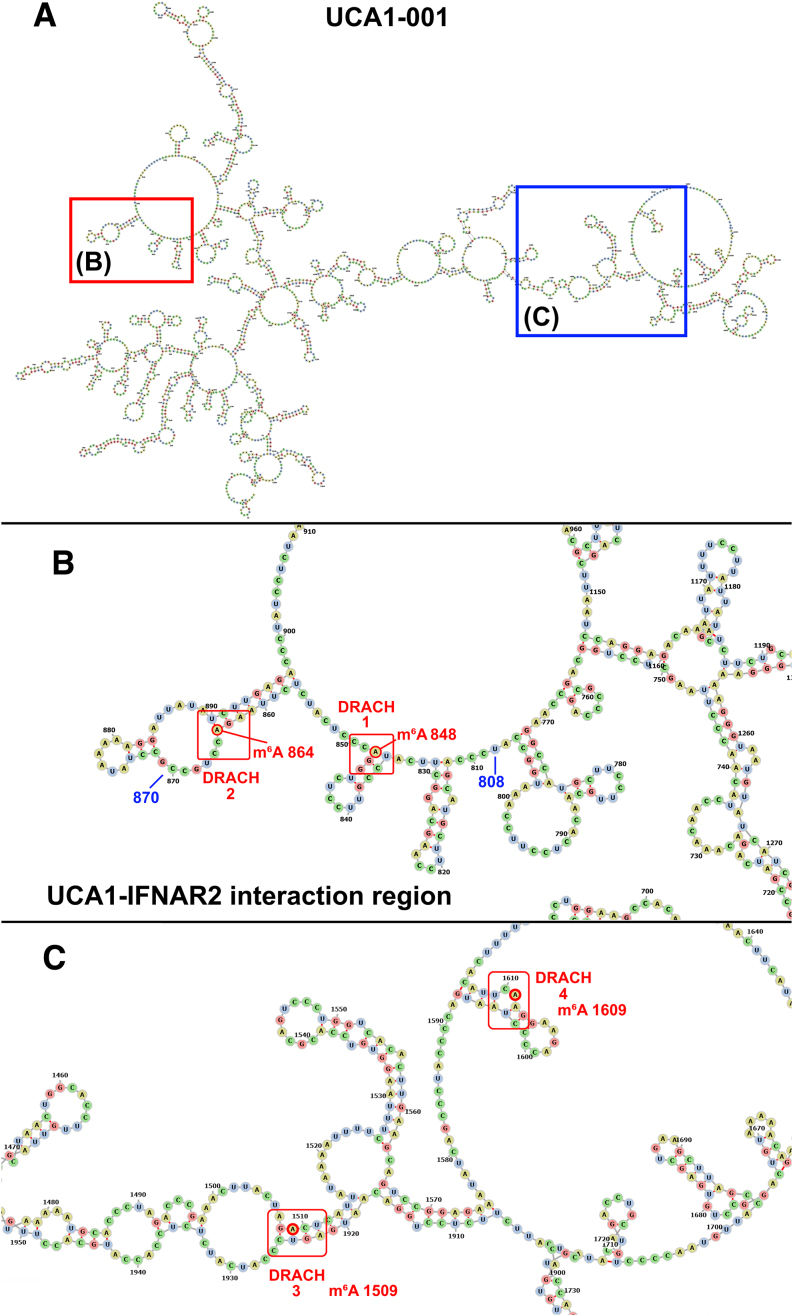
Location of DRACH motifs and m^6^A sites in the secondary structure of UCA1 lncRNA. (**A**) Minimum free energy structure of the 2229 nt transcript UCA1-001 (−563.20 kcal/mol) with hairpin loops detailed in (**B**) and (**C**). (B) Detail of the UCA1 region 808–870 that interacts with IFNAR2 mRNA. DRACH motifs 1 (GGACC) and 2 (AGACC) are in red boxes, m^6^A in 848, and 864 are indicated by red circles. (**C**) Detail of DRACH motifs with m^6^A-modified 1509 that pairs with U (DRACH 3-AGACU) and the unpaired m^6^A 1609 (DRACH 4-GAACU).

Methylated DRACH motifs were detected in the target recognition sites within UCA1 lncRNA (Fig. 4). Also, differentially methylated DRACH motifs were found in lncRNAs secondary structure regions, highlighted in UCA1 (Fig. 5). UCA1-001 (ENST00000397381) presented two methylated m^6^A sites (positions 848 and 864) within the predicted region of interaction with its target (positions UCA1-001 at 808–870 and IFNAR2-001 at 2853–2900) (Table 3 and Fig. 5). UCA1 shows m^6^A sites more methylated in infected cells, located within hairpins in the secondary structure where the m^6^A is in A-U pairs (Fig. 5). The A-U pairs in which the A is a m^6^A could form, as discussed below, noncanonical base pairs that destabilize the RNA duplexes. Differentially methylated DRACHs have also been mapped in GAS5 and NORAD secondary structures revealing a structural proximity of methylated domains (Supplementary Figs S1 and S2).

**Table 3.** Positions of DRACH motifs, m^6^A sites, and lncRNA–target RNA interactions in transcripts

LncRNA	Target RNA	LncRNA m^6^A	LncRNA–target interactions and DRACHs
GAS5	OAS1 (2′-5′-oligoadenylate synthetase 1)	448	Int.1 GAS5 1D (74–118)–OAS1 (1326–1375)
GAS5	IFN-induced protein with tetratricopeptide repeats 1	448	Int.1 GAS5 1D (57–118)–IFITI 1 (3818–3873)
GAS5	Tumor necrosis factor (TNF)	448	Int.1 GAS5 1D (88–118)–TNF (908–943)
GAS5	Toll-like receptor 9 (TLR9)	448	Int.1 GAS5 1D (93–118)–TLR9 (3801–3826)
GAS5	Signal transducer and activator of transcription 2 (STAT2)	448	Int.1 GAS5 1D (74–115)–STAT2 (983–1027)
NORAD	IFN α receptor 2 (IFNAR2)	2192, 2661	Int.5 NORAD (4104–4145)–IFNAR2 (2684–2725) 1D
NORAD	Interleukin 6 receptor (IL6R)	2192, 2661	Int.3 NORAD (4088–4146)–IL6R (2254–2324) 1D Int.5 NORAD (4090–4146)–IL6R (4090–4146) 1D
NORAD	Toll-like receptor 8 (TLR8)	2192, 2661	Int.2 NORAD (4089–4144)**–TLR8 (720–785) 2D** Int.3 NORAD (4094–4130)**–TLR8 (735–788) 2D** Int.4 NORAD (4092–4146)–**TLR8 (738–795) 2D** Int.5 NORAD (4111–4145)– TLR8 (721–758) 1D
NORAD	IFN regulatory factor 3 (IRF3)	2192, 2661	Int.2 NORAD (4090–4130)–IRF3 (2822–2863) 1D Int.3 NORAD (4100–4143)–IRF3 (2823–2873) 1D
NORAD	IFN-induced protein with tetratricopeptide repeats 1 (IFIT1)	2192, 2661	Int.3 NORAD (4102–4146)–IFIT1 (2695–2741) 1D Int.10 NORAD (4090–4135)–IFIT1(2685–2738) 1D
NORAD	Signal transducer and activator of transcription 2 (STAT2)	2192, 2661	Int.1 NORAD (4098–4147)–STAT2 (981–1025) 1D Int.2 NORAD (4090–4147)–STAT2 (981–1024) 1D Int.3 NORAD (4104–4147)–STAT2 (981–1026) 1D
NORAD	IFN α 21 (IFNA21)	2192, 2661	Int.1 NORAD (4089–4132)–IFNA21 (655–700) 1D
NORAD	IFN α receptor 1(IFNAR1)	2192, 2661	Int.8 NORAD (4087–4134)–IFNAR1 (4167–4217) 1D
NORAD	Toll-like receptor 7 (TLR7)	2192, 2661	Int.6 NORAD (4098–4152)–TRL7 (337–402) 1D
NORAD	TNF receptor-associated factor 3 (TRAF3)	2192, 2661	Int.10 NORAD (4090–4151)–**TRAF3 (6344–6395) 2D**
NORAD	Toll-like receptor 9 (TLR9)	2192, 2661	Int.9 NORAD (4090–4145)–TLR9 (1064–1122) 1D
UCA1	IFN α receptor 2 (IFNAR2)	848*, 864* 1509, 1609	**Int.7 UCA1 2D (808–870**)–IFNAR2 (2853–2900) Int.1 UCA1 1D (9–53)–IFNAR2 (2849–2899) Int.3 UCA1 1D (2024–2058)–IFNAR2 (2865–2901) Int.4 UCA1 1D (2023–2057)– IFNAR2 (2847–2880) Int.5 UCA1 1D (2024–2059)–IFNAR2 (2866–2901)
UCA1	IFN α receptor 1 (IFNAR1)	1509, 1609	Int.1 UCA1 (1997–2031)–IFNAR1 (59–91) 1D Int.2 UCA1 1D (2013–2055)–IFNAR1 (59–102) 1D
UCA1	IFN γ receptor (IFNGR2)	1509, 1609 848*, 864*	Int.1 UCA1 1D (2022–2061)–IFNGR2 (185–219) Int.7 UCA1 1D (115–149)–IFNGR2 (187–219) Int.8 UCA1 1D (19–53)–IFNGR2 (195–222)
UCA1	Mitochondrial antiviral signaling protein (MAVS)	1509, 1609 848*, 864*	Int.5 UCA1 1D (2022–2056)–MASV (11159 11186) Int.7 UCA1 1D (9–53)–MASV (11165–11197) Int.10 UCA1 1D (1997–2053)–MASV (10230–10 276)
UCA1	IFN γ receptor (IFNGR2)	1509, 1609 848*, 864*	Int.1 UCA1 1D (2022–2061)–IFNGR2 (185–219) Int.7 UCA1 1D (115–149)–IFNGR2 (187–219) Int.8 UCA1 1D (19–53)–IFNGR2 (195–222)
UCA1	Mitochondrial antiviral signaling protein (MAVS)	1509, 1609 848*, 864*	Int.5 UCA1 1D (2022–2056)–MASV (11159–11 186) Int.7 UCA1 1D (9–53)–MASV (11165–11 197) Int.10 UCA1 1D (1997–2053)–MASV (10230–10276)

Interactions (Int.) followed by the molecule (lncRNA or target) in which the DRACH was detected. Positions where m^6^A probability is within or in the vicinity of interaction region in transcripts GAS5 ENST00000430245 (GAS-027), NORAD ENST00000565493 (NORAD-001), and UCA1 ENST00000397381 (UCA1-001). Number of DRACH motifs in interaction regions, are indicated by, 1D = 1 DRACH motif and 2D = 2 DRACH motifs (bold font) and (*) indicate m^6^A methylated DRACH motifs within the interaction region, the other methylated sites are the closest to the interaction region.

**Figure 6.**
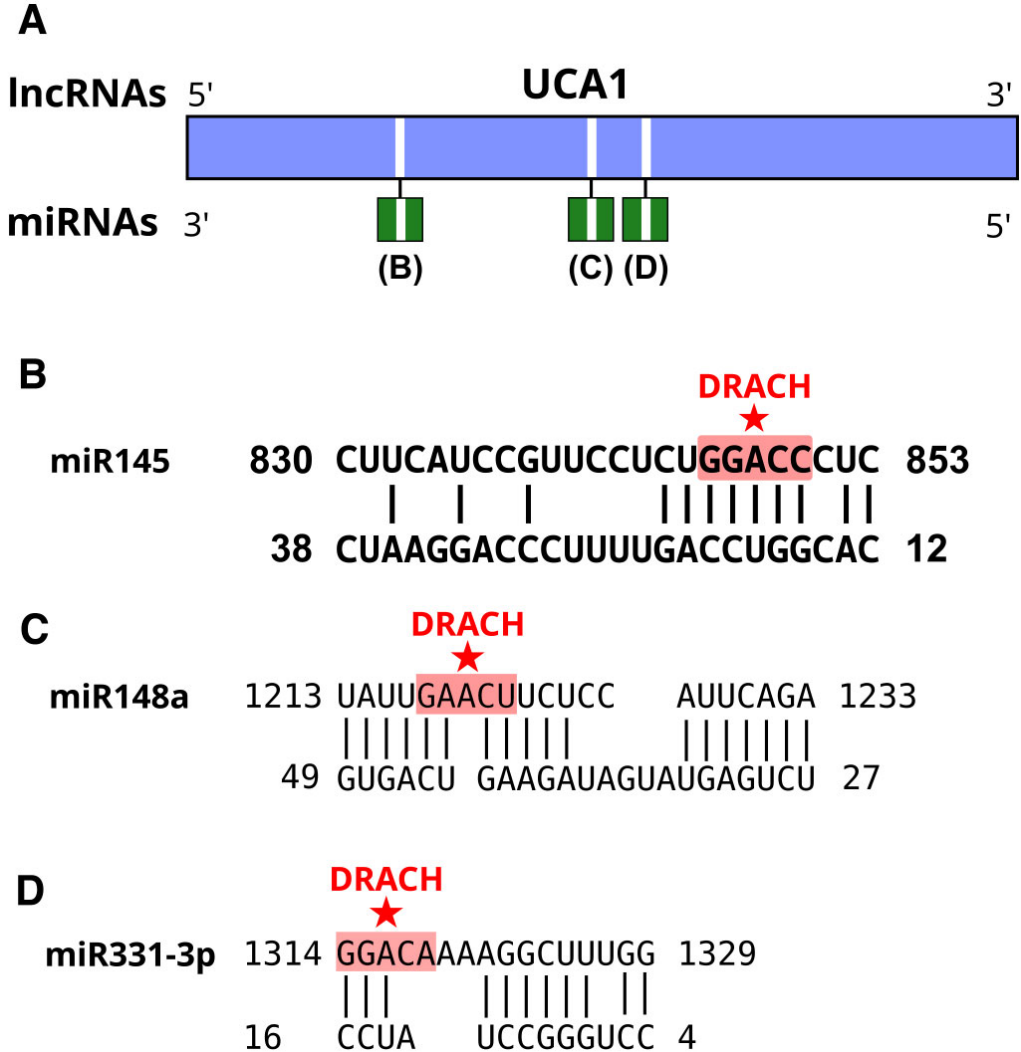
Interactions of UCA1 lncRNA with miRNAs. (**A**) Map of UCA1 interacting regions with miR145 (**B**), miR148a (**C**), and miR331-3p (**D**). The UCA1 sponging motif has been experimentally demonstrated in its interaction with miR-145 in cancer and HCV infection contexts. The star indicates the methylated m^6^A site within the DRACH motif GGACC that overlaps with the sponging motif CUGGAC. The m^6^A pairs with U which could promote transient Hoogsteen pairs and potential duplex destabilization. Interactions with miR148a and miR331-3p contain methylated DRACH motifs, but they are less stable by hydrogen bonds.

In addition to target recognition regions and secondary structures, UCA1 can function as miRNA sponge. UCA1 transcript UCA1-001 (2299 nt) contains the 5′-AGCUGGAC-3′ motif that sponges miR-145 which in turn affects STAT1 expression and impacts IFN response. Another smaller sponging motif, 5′-CUGGAC-3′, is in four positions in UCA1-001 transcript: (i) 508–513, (ii) 844–849, (iii) 1727–1732, and (iv) 1912–1917. The sponging motif 844–849 overlaps with a m^6^A methylated DRACH motif (Fig. 6B). UCA1-001 also interacts with miR148a (Fig. 6C) and miR331-3p (Fig. 6D) as detailed (Supplementary Data S1).

**Figure 7.**
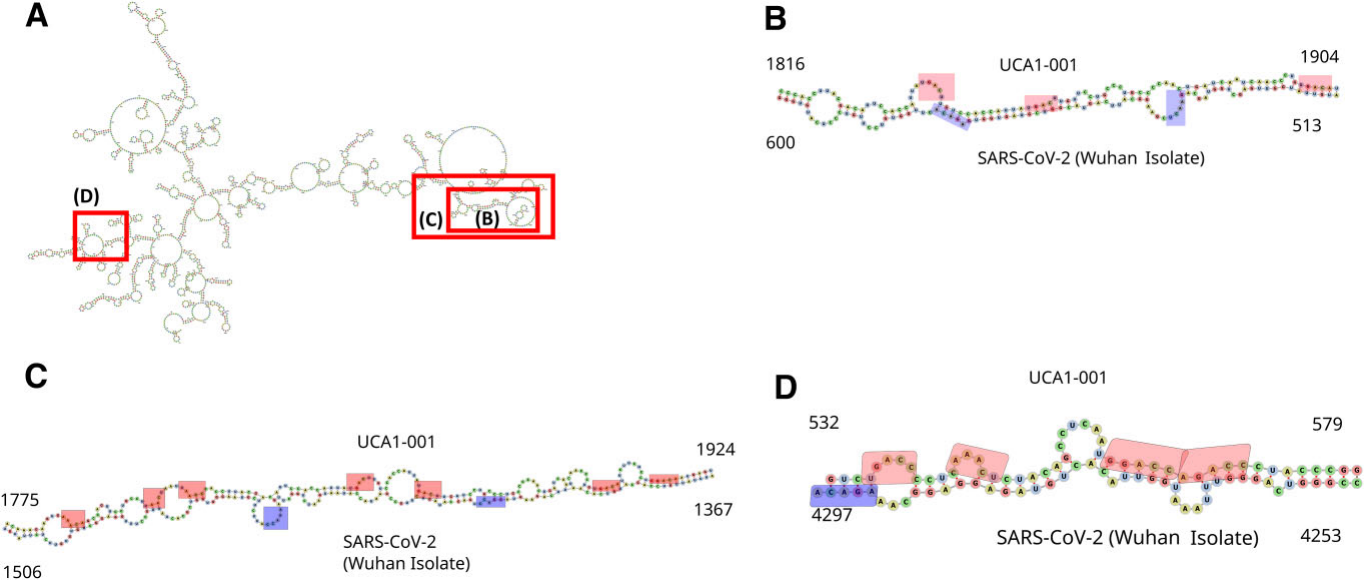
Interactions of UCA1 lncRNA with SARS-CoV-2 RNA genome. (**A**) Location of interaction regions to SARS-CoV-2 genomic RNA (Wuhan strain) in UCA1 lncRNA minimum free energy secondary structure. (**B**) UCA1 positions 1816–1904 are predicted to interact with SARS-CoV-2 RNA at positions 513–600 with energy −25.82 kcal/mol. (**C**) Interactions at UCA1 positions 1775–1941 to SARS-CoV-2 (1367–1506) with energy –21.65 kcal/mol and (**D**) UCA1 532–579 to SARS-CoV-2 4253–4297 with –24.15 kcal/mol. Light red boxes indicate DRACH motifs in lncRNA and blue boxes DRACH motifs in SARS-CoV-2 RNA. LncRNA–SARS-CoV-2 interactions were predicted using intaRNA program and confirmed by RNAhybrid program.

UCA1 is also capable of interacting with the SARS-CoV-2 genome RNA by forming strong duplexes (Fig. 7). Interactions with SARS-CoV-2 genomic RNA (Wuhan strain) can be mapped in UCA1 lncRNA minimum free energy secondary structure. UCA1 positions 1816–1904 are predicted to interact with SARS-CoV-2 RNA at positions 513–600 with energy −25.82 kcal/mol (Fig. 7B). Interactions are also predicted at UCA1 positions 1775–1941 to SARS-CoV-2 (1367–1506) with energy –21.65 kcal/mol (Fig. 7C) and UCA1 532–579 to SARS-CoV-2 4253–4297 with –24.15 kcal/mol (Fig. 7D). These interaction predictions were determined by intaRNA program and confirmed by RNAhybrid program. Interactions of UCA1 with SARS-CoV-2 Omicron were also observed and mapped to the same regions with equivalent energies despite minimal sequence variations (Supplementary Fig. S3 and Supplementary Data S2).

### Expression of lncRNAs in COVID-19 patients

The expressions of lncRNAs UCA1, GAS5, NORAD, BISPR, and NEAT1 were quantified using Illumina cDNA (RNA-seq) sequencing data and compared to dRNA-seq using fold change expression in infection (normalized reads in infected/normalized reads in uninfected) (Supplementary Table S7). The upregulation observed of GAS5, NORAD, BISPR, and NEAT1 in dRNA-seq of infected Calu-3 cells (between 1.03- and 1.11-fold) is consistent with upregulation of these lncRNAs in COVID-19 patients (1.05- and 1.17-fold) in the two datasets (GSE171110 and GSE157103). UCA1, however, is upregulated in patients (1.24- and 1.63-fold) as opposed to the observed downregulation in 48 h infected Calu-3 cells (0.84-fold) (Supplementary Table S7). The expressions of IFN and IFN receptors were also quantitated in transcriptome data of COVID-19 patients (Supplementary Table S8). The whole blood transcriptome shows that IFN and IFN receptors are upregulated in a similar fashion to observed in Calu-3-infected cells (Table 6).

## Discussion

### Global and local m^6^A changes in lncRNAs

The central idea of this study is to demonstrate that m^6^A methylation of lncRNAs is altered by SARS-CoV-2 infection. While global m^6^A remodeling of host mRNAs during SARS-CoV-2 infection has been previously reported, analogous studies of lncRNAs are lacking. Unlike coding transcripts, lncRNAs often act via structure-dependent interactions, making their methylation patterns—and the structural consequences thereof—particularly relevant.

We investigated the expression of 10 selected lncRNAs, from an original curated set of 100 lncRNAs known to be associated with SARS-CoV-2 infection, particularly on the potential to modulate the host’s immune response, and viral replication. We observed a tendency of increased expression of these 10 lncRNAs in SARS-CoV-2-infected Calu-3 cells, except for the downregulated UCA1, suggesting a coordinated activation of these transcripts as part of the host’s intracellular antiviral response (Fig. 1). Also, the global pattern of increased lncRNA m^6^A methylation in infected cells, is consistent with increased m^6^A methylation in mRNAs, as previously observed [5] (Fig. 2). Although a global trend of increased lncRNA m^6^A methylation is statistically supported, given the lncRNA set considered (60% m^6^A probability cutoff), we observed that m^6^A methylation of each individual lncRNA and DRACH motif may increase, decrease or remain stable upon infection, depending on the context of each individual lncRNA and individual DRACH motif within each lncRNA, which we evaluated in detail (Tables 2 and 3, and Fig. 2). The expression profiles of the 10 lncRNAs detailed in Table 1 were estimated from dRNA-seq data by normalization of reads mapped to the lncRNA reference sequences (Fig. 1). Most of the selected lncRNAs displayed modest upregulation upon infection, with fold changes ranging from 1.03 to 1.06 (~3%–6%). Small variations in lncRNA abundance can tip the balance of competing endogenous RNA (ceRNA) networks, derepressing or repressing whole sets of mRNAs [50]. lncRNAs are often lowly expressed and therefore a 3%–6% change in a low-copy transcript can correspond to a handful of molecules per cell, which may be enough to alter binding equilibria with RBPs or miRNAs [51–53]. Transcription factors and miRNAs often show modest fold changes that drive major transcriptional or phenotypic shifts as in the role of miR-1/miR-133 in muscle differentiation [54]. Regulatory networks are characterized by nonlinear biological response which can amplify small upstream changes into large downstream responses (signal amplification, bistability, and feedback loops) [55]. These upregulation levels observed in dRNA-data were also observed in independent cDNA sequencing with illumina technology in COVID-19 patient transcriptome data (Supplementary Table S7).

### Expression levels and m^6^a patterns of lncRNAs and IFN response

According to changes in the expression levels of individual lncRNAs and their m^6^A methylation patterns we predict the outcome in terms of the IFN (INF) response as summarized in Table 5. Our analysis reveals distinct patterns of expression and m^6^A methylation among key lncRNAs in SARS-CoV-2 infection (Table 2; Supplementary Tables S3–6). Several lncRNAs with established or putative roles in IFN signaling exhibit coordinated changes in expression and m^6^A modification. UCA1 showed decreased expression but increased m^6^A methylation. Previous studies indicate that m^6^A on UCA1 reduces its ability to sponge miR-145-5p, thereby lifting repression on *SOCS7*, a known inhibitor of IFN signaling [56]. This mechanism could result in suppressed IFN responses despite viral infection, aligning with known viral strategies to evade immune detection (Table 5). The simultaneous downregulation of UCA1 may reinforce this suppressive effect by reducing the lncRNA pool available for miRNA interaction. NORAD and GAS5 both exhibited increased expression and increased m^6^A levels. In NORAD, m^6^A has been shown to reduce sequestration of Pumilio RNA Binding Family Member (PUM1), a post-transcriptional repressor of ISGs. This would enhance PUM1’s ability to degrade ISG transcripts, ultimately weakening the IFN response [57] (Table 5). Similarly, in GAS5, m^6^A appears to reduce its sponging capacity for miRNAs like miR-21, which could diminish STAT1 activity and thereby attenuate IFN expression [58] (Table 5). These patterns point to a broader trend in which m^6^A may facilitate viral immune evasion by functionally impairing lncRNA-mediated regulatory mechanisms, such as the IFN response.

In contrast, BISPR displayed increased expression but reduced methylation (Table 2). BISPR is a positive regulator of *BST2*, an antiviral factor. Hypomethylation may enhance BISPR stability or function, thus promoting *BST2* expression and activation of IFN responses [59]. This is one of the few examples in our dataset where a methylation change could support, rather than inhibit, antiviral immunity (Table 5). CASC2, while upregulated, did not exhibit a significant change in m^6^A methylation, and its role in the IFN pathway remains uncertain [60] (Table 5). Similarly, MIR155HG and THRIL were upregulated without corresponding changes in methylation, suggesting that their regulatory roles—such as miR-155 biogenesis and modulation of TNFα/IFNβ expression—remain intact and are likely governed by transcriptional or post-transcriptional dynamics unrelated to m^6^A [61, 62] (Table 5). MIR155HG is a precursor of miR-155, a key regulator of innate immune response associated with hyperinflammatory conditions in COVID-19 [20].

LINC00278 and LINC01619 are both upregulated and hypermethylated but have ambiguous roles. LINC00278 encodes a micropeptide, and its m^6^A modification may influence translation or RNA–RNA interactions, though its impact on the IFN pathway appears context-dependent [63] (Table 5). For LINC01619, structural or interaction-based modulation by m^6^A is plausible, but the relevance to antiviral immunity has yet to be clarified [64] (Table 5). LINC00511 is upregulated but hypomethylated and may exert increased repressive effects on the IFN pathway if m^6^A typically constrains its function. This raises the possibility that demethylation could enhance its negative regulatory role under viral infection [65] (Table 5).

NEAT1 [66] expression is elevated in saliva and nasopharyngeal samples of COVID-19 patients [67] and upregulated in lung epithelial cells [68]. The dRNA-seq here analyzed shows, however, that NEAT1 is downregulated in infected Calu-3 cells (FC = 0.72) (Supplementary Table S2). Mapping of dRNA-seq reads to NEAT1 identified DRACH motifs and a predicted m^6^A, >60% probability, only in position 16 648 with modest number or reads as compared to downstream sites (Supplementary Table S6). This suggests that the NEAT1 transcript present in Calu-3 cells comprises exons located near the 3′ terminal region (approximately from positions 16 000 to 18 000) of the NEAT1 gene and not the ENSEMBL canonical 22k nt form (https://www.ensembl.org/). We also examined the location of this methylated site within predicted RNA secondary structures. These results suggest that NEAT1 may be subject to isoform-specific and structure-informed m^6^A regulation in SARS-CoV-2-infected cells. Nevertheless, the combined effect of downregulated NEAT1 and low m^6^A methylation predicts an outcome of a weakened type I IFN response, potentially contributing to viral immune evasion (Table 5).

To compare the expression of lncRNAs UCA1, GAS5, NORAD, BISPR, and NEAT1 in the initial cellular phase of SARS-CoV-2 infection with the systemic level observed in COVID-19 patients these lncRNAs were quantified on Illumina cDNA (RNA-seq) sequencing data and compared to dRNA-seq data (Supplementary Table S7). Epitranscriptome data by dRNA-seq is not available for COVID-19 patients. The observed upregulation of GAS5, NORAD, BISPR, and NEAT1 in dRNA-seq of infected Calu-3 cells (1.03- and 1.11-fold) is consistent with upregulation of these lncRNAs in COVID-19 patients (1.05- and 1.17-fold) in the two datasets (GSE171110 and GSE157103). UCA1, however, is upregulated in patients as opposed to the observed downregulation in 48 h infected Calu-3 cells (Supplementary Table S7). The slightly higher upregulation of GAS5, NORAD, BISPR, and NEAT1 as compared to dRNA-seq data might reflect the difference between the initial cellular level events of SARS-CoV-2 infection detected by dRNA-seq as opposed to the systemic, inflammatory multicellular events detected by cDNA-seq (RNA-seq) Illumina sequencing, which was obtained from RNA extracted from whole blood. Both dRNA-seq and cDNA-seq show that the overall level of UCA1 is low. The upregulation of UCA1 in patients as opposed to initial single cell type cellular events might also reflect differences in initial infection events as opposed to systemic later stages of infection. Expression of IFN and IFN receptors were upregulated both in Calu-3 cells and patents (Supplementary Table S8) which shows that initial infection and later systemic disease behave in similar fashion regarding these genes.

Our results provide a conceptual model based on lncRNA expression/methylation with testable predictions on IFN response. Accordingly, we examined the IFN expression profiles from the same dRNA-seq dataset, used for quantification lncRNA expression and m^6^A methylation, to test the anti-viral response predicted by our model (Table 5). We show that transcripts for IFN-α (IFNA1, IFNA2), and IFN-γ (IFNG) were undetectable in both conditions, echoing reports of suppressed or delayed type I and II IFN responses in SARS-CoV-2–infected cells [69] (Table 6). Also, our data shows a selective induction of IFN-β (IFNB1), with 77 reads detected in infected cells versus none in uninfected cells, corresponding to a 68.55-fold change (Table 6). Although the absolute read count is modest, this finding is consistent with prior studies reporting low but inducible *IFNB1* expression in epithelial cells upon viral infection [70]. Receptors for type I and II IFNs (IFNAR1, IFNAR2, IFNGR1, and IFNGR2) showed modest upregulation (fold changes ∼1.03–1.04), suggesting preserved or slightly enhanced signaling capacity. Together, these data align with a controlled, IFN-β-centered antiviral response and support our hypothesis that epitranscriptomic remodeling—including m^6^A modifications in lncRNAs—may fine-tune IFN signaling. Specifically, m^6^A enrichment in functionally relevant regions of lncRNAs such as UCA1, NORAD, and GAS5 may modulate their interactions with immune-related mRNAs, miRNAs, or RBPs, contributing to the specificity and magnitude of the host response.

### LncRNA m^6^A methylation and interactions with miRNAs

Several lncRNAs sponge miRNAs and lncRNA–miRNA interactions involve base pairing [71]. For example, GAS5 sponges miR21 which in turn regulates the expression of STAT1, an activator of IFN transcription [72]. The sequencing data here analyzed, however, does not contain any miRNAs because the default read size cutoff of Nanopore sequencing excludes reads smaller than 200 nt. We attempted to detect miRNA in this dRNA-seq dataset using Minimap2, but no reads mapped to miRNA references with statistical significance. Therefore, we could not investigate whether miRNAs contain methylated DRACH motifs with the data at hand. This additional layer of gene regulation, involving the potential effects of m^6^A in miRNAs and the interaction to lncRNAs [73], remains an important question to be investigated (Table 7).

According to current scientific literature, UCA1 has not been reported to play a role in SARS-CoV-2 (COVID-19) infections. Instead, its involvement is documented in other contexts, such as cancer, sepsis, acute respiratory distress syndrome unrelated to COVID-19, and infection by other viruses like hepatitis C. UCA1 is particularly interesting because (i) its known regulatory role in viral infections, (ii) its altered expression in SARS-CoV-2 infection, (iii) the presence of multiple m^6^A-modified DRACH motifs, and (iv) its predicted interactions with miR-145 and SARS-CoV-2 RNA (Table 7). These features make it a representative model for exploring m^6^A-guided lncRNA–miRNA–viral RNA interactions. Experimental evidence shows that sponging of miR-145 by UCA1 is dependent on sequence 5′-AGCUGGAC-3′ in UCA1 transcript EU334869.1 (ENST00000644174.2 UCA1-213 with 2688 nt) that forms a duplex with miR-145 sequence 3′-UUGACCUG-5′ in gastric cancer [74]. This sponging motif, present in UCA1 transcript UCA1-213, is present in UCA1-001, here analyzed, at position 506–513. This motif does not contain a DRACH motif although it is close to the methylated m^6^A in DRACH in position 663. Therefore, UCA1-001 with 2299 nt contains the 5′-AGCUGGAC-3′ motif and is predicted to sponge miR-145 which in turn affects STAT1 expression and impacts IFN response. How the differential m^6^A methylation of position 663 affects the sponging of miR-145 is to be tested experimentally. In HCV infection, UCA1 sponges miR-145-5p which regulates the level of the suppressor of cytokine signaling 7 (SOCS7), and in turn regulates the antiviral response in Huh7.5 cells [56]. The UCA1 motif that mediates this sponging is 5′-CUGGAC-3′ that locates in four positions in UCA1-001 transcript; (i) 508–513, (ii) 844–849, (iii) 1727–1732, and (iv) 1912–1917. The sponging motif 844–849 overlaps with a m^6^A methylated DRACH motif (Fig. 6). This intriguing observation offers an explicit hypothesis to be tested experimentally in future studies via genetic modification of Calu-3 cells with mutants of this sponge motif and their phenotypic changes in SARS-CoV-2 infection.

### LncRNA interactions with SARS-CoV-2 RNA

Location of interaction regions to SARS-CoV-2 genomic RNA (Wuhan strain) in UCA1 lncRNA minimum free energy secondary structure. UCA1 positions 1816–1904 are predicted to interact with SARS-CoV-2 RNA at positions 513–600 with energy −25.82 kcal/mol. Interactions at UCA1 positions 1775–1941 to SARS-CoV-2 (1367–1506) with energy −21.65 kcal/mol. UCA1 532–579 to SARS-CoV-2 4253–4297 with −24.15 kcal/mol. Light red boxes indicate DRACH motifs in lncRNA and blue boxes DRACH motifs in SARS-CoV-2 RNA. LncRNA–SARS-CoV-2 interactions were predicted using intaRNA program and confirmed by RNAhybrid program (Fig. 7). This region contains three DRACH motifs, although not methylated as indicated by in our analysis using m6anet. This unmethylated status may result from structural occlusion caused by early or persistent base-pairing with viral RNA, preventing access of the METTL3/METTL14 complex. Alternatively, the absence of m^6^A may facilitate hybridization, because methylation typically disrupts base pairing and promotes more open conformations. Both scenarios are consistent with the observed downregulation of UCA1 in infected cells and support a model in which the virus-induced RNA–RNA interaction alters the methylation landscape and post-transcriptional fate of host lncRNAs.

### LncRNAs and signaling pathways

NORAD [46], THRIL [47], and GAS5 [43] are involved in regulation of the NF-κB signaling pathway, a central axis of immune activation in response to respiratory viral infections and showed increased expression in infection (Fig. 1). Although activation of the NF-κB pathway is essential for controlling viral infections, its excessive activation is associated with systemic inflammation and worse clinical outcomes in severe cases of COVID-19 [75]. Therefore, post-transcriptional and epigenetic regulation of lncRNAs such as GAS5 and NORAD may represent a compensatory mechanism of immune modulation, to balance antiviral defense and prevent tissue damage due to excessive inflammation. GAS5 acts as sponge, inhibiting miRNAs that repress components of the NF-κB pathway and amplify the inflammatory response [43]. NORAD interacts with PUMILIO proteins (PUM1 and PUM2), key post-transcriptional repressors that bind to target mRNAs and promote their degradation and translational silencing. By sequestering PUMILIO proteins, NORAD prevents the repression of genes involved in DNA repair, stress responses, and cell cycle regulation [46]. In the context of SARS-CoV-2 infection, this regulatory mechanism may contribute to genomic stability and modulate inflammatory responses. THRIL directly modulates TNF-α transcription by forming a complex with hnRNPL, a factor involved in inflammatory response [47]. Therefore, the increased expression of these lncRNAs may play an active role in amplifying or modulating the cytokine-mediated inflammatory response during SARS-CoV-2 infection.

CASC2 and LINC01619 are downregulated in viral infections [44, 49]. However, these two lncRNAs show increased expression in SARS-CoV-2 infection as estimated from dRNA-seq data here analyzed (Fig. 1). This suggests potential changes in RNA stability, alternative splicing, or other post-transcriptional mechanisms induced by viral stress. We also observed that most of the lncRNAs are *trans*-acting regulators, in line with their previously described roles in the regulation of multiple cellular pathways [19]. An exception is BISPR, a *cis*-acting regulator of ISG *BST2* (tetherin), whose coordinated co-expression was reported in COVID- 19 patients [20], reinforcing its role in the IFN-mediated antiviral response. In viral infection, *trans*-acting lncRNAs can regulate processes ranging from ISG expression to redox homeostasis and apoptosis [18].

Our analysis shows increased m^6^A methylation of GAS5, NORAD, and UCA1 during infection, pointing to further layers of post-transcriptional regulation [48]. The observed loss of the m^6^A modification in BISPR post-infection (Table 2) may indicate a decrease in m^6^A-mediated decay, therefore enhancing transcript stability and enabling its upregulation and *cis-*mediated regulation of *BST2*. In contrast, lncRNAs LINC00278 and NORAD acquired novel m^6^A sites post-infection, suggesting a potential role for m^6^A in stress-induced activation or stabilization. UCA1 functions as a molecular sponge for pro-inflammatory miRNAs, such as, miR-143, miR-204, and miR-27b, involved in regulation of cell proliferation, apoptosis, survival and modulate *FOXO1, BCL2*, and PI3K/AKT pathway, under cellular stress and viral inflammation [76–78]. Therefore, the acquisition of novel m^6^A sites may enhance transcript stability and indirectly promote the translation of target genes to alter the inflammatory response. In our study, UCA1 exhibited the highest number of m^6^A sites with high modification probability (>90%) compared to other lncRNAs analyzed, indicating an intense epitranscriptome activity in SARS-CoV-2 infection. The presence of multiple highly methylated residues indicates that UCA1 is a preferential target of m^6^A methylation machinery. This is supported by the demonstration of targeted recruitment of METTL3/14 to specific regulatory RNAs under stress and viral infection, resulting in functional alterations in the stability and translation of these RNAs [79, 80].

### DRACH motif nucleotide bias

DRACH motif analysis revealed subtle alterations in sequence context, which may reflect increased substrate specificity of the methyltransferase complex under infection conditions (Fig. 3 and Table 4 ). Although the GAC core is conserved, an enrichment of A/U nucleotides was observed in the flanking regions in infected cells, particularly among lncRNAs with a higher number of variant m^6^A sites. This shift in sequence profile may reflect a reprogramming of methyltransferase substrate specificity, favoring m^6^A methylation in more permissive sequence contexts. Such changes may be linked to alterations in the activity of cofactors such as WTAP [4], and modulation of DRACH preferences by SARS-CoV-2 itself that suggests a viral adaptation mechanism aimed at exploiting the host’s epitranscriptome machinery [6].

**Table 4.** Summary table of the most and second-most frequent nucleotides at each position in the DRACH motif flanking m^6^A sites in each lncRNA under uninfected and infected conditions

lncRNA	Condition	−2	−1	0 (m^6^A)	+1	+2
UCA1	Uninfected	G/a	G	A	C	A/c
UCA1	Infected	G/u	G	A	C	A/u
NORAD	Uninfected	A/g	A/g	A	C	U
NORAD	Infected	G/A	G	A	C	A/U
GAS5	Uninfected	U/a	G	A	C	A/c
GAS5	Infected	U/a	G	A	C	A/c
LINC01619	Uninfected	A	G	A	C	A/U
LINC01619	Infected	A	G	A	C	U

The methylated base (A) is located at position 0. For each position, the dominant base is shown in uppercase; the second-most frequent base is shown in lowercase, separated by a slash. If two bases were equally frequent, both are shown in uppercase (e.g. A/U). Notable infection-associated shifts include the +2 position in UCA1 (A/c → A/u) and NORAD (U → A/U), suggesting infection-specific alterations in m^6^A sequence context.

**Table 5.** Prediction of IFN response as effect of lncRNAs expression (Exp.) and m^6^A methylation in SARS-CoV-2 infection

LncRNA	Exp.	m^6^A	Proposed mechanism	IFN	Ref.
UCA1	↓	↑	m^6^A reduces miR-145–5p binding → ↑ SOCS7 → suppresses IFN signaling	↓	[56]
NORAD	↑	↑	m^6^A reduces PUM1 sequestration → ↑ PUM1-mediated decay of ISG mRNAs	↓	[57]
GAS5	↑	↑	m^6^A reduces miRNA sponging (e.g. miR-21) → ↓ STAT1 expression	↓	[58]
BISPR	↑	↓	Reduced methylation enhances lncRNA stability/function → ↑ BST2 expression → activates IFN response	↑	[59]
CASC2	↑	=	No change in m^6^A; IFN-modulatory role remains unclear	=	[60]
LINC00278	↑	↑	^6^mA may affect translation of encoded micropeptides or RNA–RNA interactions; role in IFN unclear	Context-dependent	[63]
LINC00511	↑	↓	Reduced methylation may enhance repressive function on IFN pathway	↓ (if repressor)	[65]
LINC01619	↑	↑	m^6^A likely affects structure or interactions; IFN pathway relevance not yet known	Unknown	[64]
MIR155HG	↑	=	Precursor of miR-155; no m^6^A change → likely unchanged miRNA processing or IFN effects	=	[61]
THRIL	↑	=	Known modulator of TNFα and IFNβ; unchanged m^6^A suggests conserved regulatory role	=	[62]
NEAT1	↑	↓	↓NEAT1 stability + ↓nuclear retention → ↓paraspeckles → less regulation of IRF7, RIG-I	↓	[81]

**Table 6.** Expression of IFNs α, β and γ and respective receptors in uninfected and SARS-CoV-2-infected Calu-3 cells

IFN/Receptor	Reads	Fold change
**IFN-α (*IFNA1***)		**1**
Uninfected	0	
Infected	0	
**IFN-α (*IFNA2***)		**1**
Uninfected	0	
Infected	0	
**IFN-β (*IFNB1***)		**68.55**
Uninfected	0	
Infected	77	
**IFN-γ (*IFNG***)		**1**
Uninfected	0	
Infected	0	
**IFNAR1 (IFN-α/β receptor subunit 1**)		**1.03**
Uninfected	14 996	
Infected	17 393	
**IFNAR2 (IFN-α/β receptor subunit 2**)		**1.04**
Uninfected	12 646	
Infected	14 815	
**IFNGR1 (IFN-γ receptor 1**)		**1.04**
Uninfected	4 013	
Infected	4 679	
**IFNGR2 (IFN-γ receptor 2**)		**1.03**
Uninfected	16 603	
Infected	19 201	

The expression of IFNs and their receptors in infected cells (fold-change) inferred from dRNA-seq reads mapped to corresponding IFN and IFN receptor reference sequences.

**Table 7.** Immunoregulatory interactions of lncRNAs GAS5, NORAD, UCA1 and NEAT1 as sponges of key miRNAs

lncRNA	Target miRNA	Function in the immune response	Reference
**GAS5**	miR-21	GAS5 acts as miR-21 sponge, promoting apoptosis and suppressing inflammation and cell proliferation, thus modulating immune responses	[82]
	miR-23a-3p	GAS5 functions as molecular sponge for miR-23a-3p, promoting inflammation and apoptosis through the upregulation of TLR4 expression	[83]
	miR-222	GAS5 inhibits miR-222, reducing PI3K/Akt activation and modulating inflammatory responses	[84]
	miR-103	By inhibiting miR-103, GAS5 promotes PTEN expression and contributes to the regulation of apoptosis and inflammatory responses	[85]
	miR-155–5p	GAS5 sponges miR-155–5p,upregulates SIRT1 and suppressing inflammation	[86]
**NORAD**	miR-155–5p	By sponging miR-155–5p, NORAD enhances SOCS1 expression and modulates cytokine signaling via the JAK/STAT pathway	[87]
	miR-485	NORAD suppresses inflammation by miR-485 sponging and enhancing NRF1 expression, contributing to antiviral immune regulation in SARS-CoV-2 infection	[88]
	miR-346	NORAD may modulate immune responses by sponging miR-346 and regulating NF-κB, DNA repair, and inflammation-related genes	[89]
**UCA1**	miR-148a	UCA1 sponges miR-148a, upregulating PD-L1 and suppressing CD8⁺ T-cell cytotoxicity	[90]
	miR-331–3p	UCA1 sponges miR-331–3p,upregulating BRD4 and enhancing pro-inflammatory cytokine production	[91]
	miR-145	UCA1 sponges miR-145 regulating myosin VI (MYO6) in gastric cancer	[74]
**NEAT1**	miR-374b-5p	NEAT1 sponges miR-374b-5p, reduces IL-6 expression and dampens inflammation in COVID-19 patients	[92]
	miR-155–5p	NEAT1 sponges miR-155–5p, increasing SOCS1 expression and inhibiting NF-κB-mediated inflammation	[93]
	miR-146a	NEAT1 regulates miR-146a, modulating the TLR4/NF-κB signaling pathway and the production of inflammatory cytokines	[94]

### Intra- and inter-molecular duplexes of lncRNAs and noncanonical base pairs

The present analysis shows the enrichment of m^6^A sites in intra-molecular duplexes, such as lncRNA secondary structure hairpins, and one in an inter-molecular RNA duplex (UCA1–IFNAR2) (Figs 4 and 5). These modifications coincide with observed changes in expression levels of known lncRNA targets, suggesting that the m^6^A modifications interfere with regulatory activity of lncRNAs [95]. Notably, among the lncRNAs analyzed, only UCA1-001 (ENST00000397381) presented two methylated m^6^A sites (positions 848 and 864) within the predicted region of interaction with its target (in UCA1-001 at 808–870 and IFNAR2-001 at 2853–2900) (Table 3 and Fig. 4). This is an exception to the general pattern we observed: lncRNA–target interaction sites tend to avoid DRACH motifs and m^6^A methylation, suggesting a selective pressure against direct methylation in these regions (Table 3). Intriguingly, in the interaction regions when a DRACH motif is present in the lncRNA, the corresponding target lacks one, and vice versa, except for UCA1 interaction (positions 2013–2055) with IFNAR1 (positions 59–102) (Table 3). Rather than acting directly at the interaction interface, m^6^A modifications appear to exert their effects by modulating the secondary or tertiary structures of lncRNAs [96]. These structural changes could influence target recognition by altering accessibility and binding. Changes in lncRNA structure associated with altered m^6^A sites can also impact the accessibility to reader proteins that mediate RNA stability. Therefore, no single, overarching rule governs the presence of m^6^A within RNA interaction regions, which suggests a regulatory trend mediated by context-dependent structural modulation, in keeping with the dynamic nature of lncRNA function.

**Figure 8.**
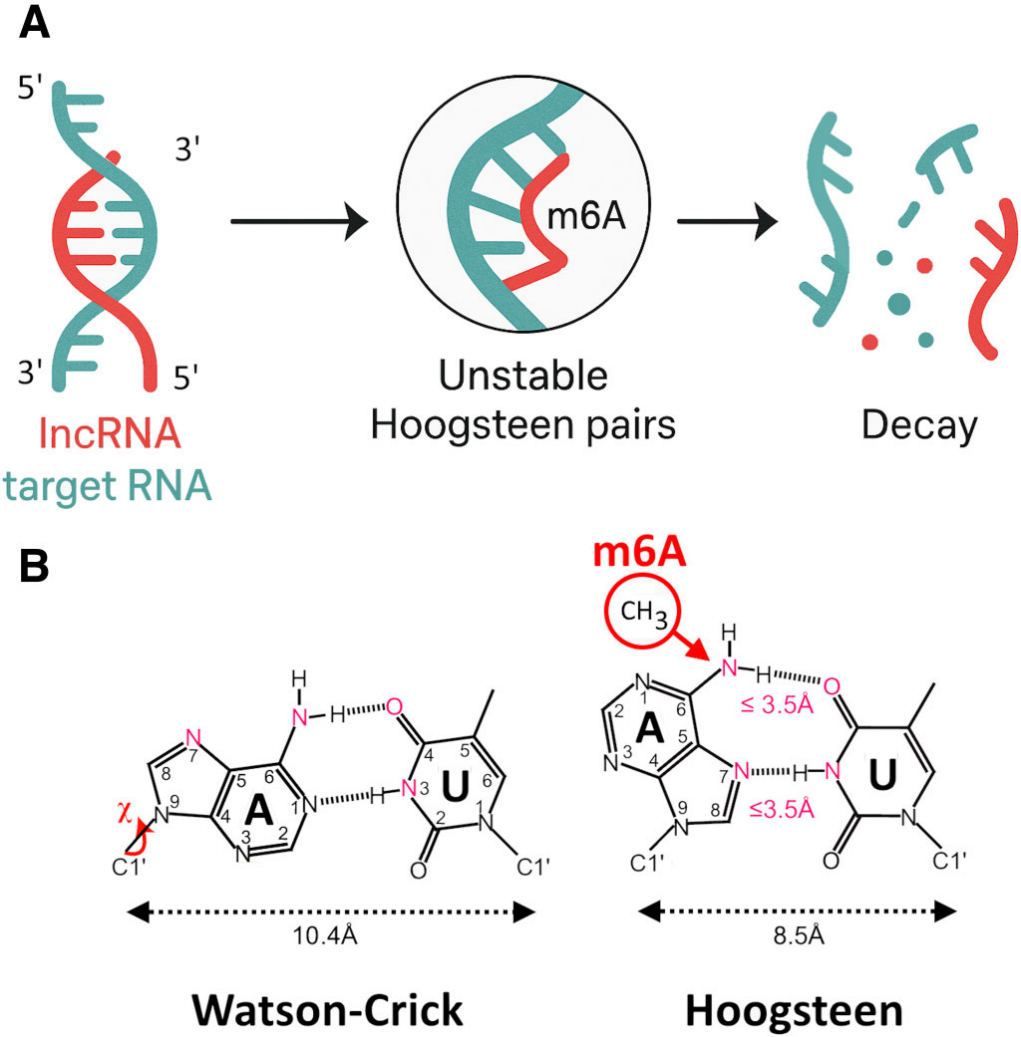
RNA positions with m^6^A and Hoogsteen base pairing. In (**A**) the target sequence, and surrounding bases, in the target RNA or the target recognition sequence in the lncRNA can be modified by m^6^A because they contain DRACH motifs. In (**B**) m^6^A methylation can increase the probability of Hoogsteen pairs that turns the hybridization unstable leading to degradation. The addition of a methyl group at the N6 position causes the torsion (χ) of the adenine (**A**) and reduces the width of the RNA–RNA hybrid from 10.4 to 8.5 Å causing its instability. (**B**) Modified from [98].

One potential molecular mechanism underlying this phenomenon involves the destabilization of lncRNA duplexes via m^6^A-dependent interference with base pairing. It is well-established that m^6^A disrupts canonical Watson–Crick A-U base pairing by introducing steric hindrance at the *N*^6^ position of adenosine, thereby lowering the thermodynamic stability of RNA duplexes [97]. Beyond this destabilization, recent structural studies suggest that m^6^A may promote the transient formation of Hoogsteen-like base pairs [95, 98]. Although Hoogsteen conformations are generally disfavored in A-form RNA helices, their transitory occurrence may contribute to local instability within RNA–RNA interaction interfaces. In the context of lncRNA–target recognition, m^6^A-induced switching between canonical and noncanonical base-pairing geometries could reduce the effective residence time of lncRNAs on their mRNA or miRNA targets, weakening or abolishing regulatory interactions. We propose that m^6^A methylation destabilizes canonical A–U base-pairing in hairpins and RNA–RNA duplexes, enabling transient Hoogsteen-like base-pairing. This conformational switch could reduce the stability of lncRNA–target hybrids, altering regulatory efficiency. Such structural shifts may explain the concordant changes in lncRNA methylation and gene expression we observed in SARS-CoV-2-infected cells (Fig. 8). The noncanonical base pairings render the RNA–RNA hybrids more unstable especially in positions involved in hairpin structures (Fig. 5). m^6^A disrupts Watson–Crick A-U pairing and can transiently switch to Hoogsteen A-U pairing in A-rich loops [95] in RNA–RNA hybrids [25] (Fig. 8). The m^6^A–Hoogsteen model offers a unifying hypothesis linking lncRNA methylation shifts to altered gene expression, opening new avenues to explore how RNA modifications dynamically modulate RNA–RNA interactomes during viral infection.

Interestingly, among most lncRNA–target interaction regions analyzed, m^6^A-modified DRACH motifs were present but did not participate in base pairing with the complementary strand (Fig. 4, Fig. 5). This suggests that these motifs, and the associated m^6^A sites, may be functionally tolerated in other duplex regions but are generally avoided within lncRNA interaction sites. While not conclusive, this observation is consistent with the idea that lncRNA–target interactions may disfavor m^6^A-modified regions, or alternatively, that m^6^A sites are selected against in inter-molecular duplex-forming domains. Notably, DRACH motifs and m^6^A modifications were abundant elsewhere in the lncRNA molecule, suggesting a potential spatial segregation between interaction domains and methylation sites.

This study presents a computational and theoretical analysis based on dRNA-seq data, aiming to generate testable hypotheses on the epitranscriptomic regulation of lncRNAs during SARS-CoV-2 infection. While experimental validation—such as reporter assays or RNA immunoprecipitation—is essential to confirm these predictions, such experiments are planned for future work and fall beyond the scope of the present manuscript.

## Conclusion

Mapping m^6^A methylation patterns using dRNA-seq data enables single-base resolution of RNA modifications in native molecules without the need for conversion, amplification or antibodies [5, 99]. Remodeling of the host epitranscriptome is documented in mRNAs during SARS-CoV-2 infection [100] and later validated in immunological models [101]. By integrating expression profiling, single-nucleotide resolution m^6^A mapping, and DRACH motif analysis in a human pulmonary cell model, we suggest that lncRNAs such as GAS5, NORAD and UCA1 are transcriptionally responsive and preferential targets of the m^6^A machinery. We propose a theoretical model in which SARS-CoV-2 induces m^6^A remodeling of lncRNA. Our analysis suggests that UCA1, particularly, plays a relevant role in SARS-CoV-2 infection response via m^6^A methylation. The m^6^A remodeling in lncRNAs may impact their ability to bind and regulate target RNAs. Transient, unstable Hoogsteen-like base pair geometries can be formed at critical RNA duplex regions that contain m^6^A which possibly affect the stability of RNA–RNA interactions and modify gene expression patterns associated with IFN response. To our knowledge, this is the first comprehensive study to characterize m^6^A dynamics of lncRNAs in SARS-CoV-2 infection in a physiologically relevant *in vitro* model. These findings extend the understanding of host–virus interactions at the epitranscriptome level and highlights the potential of m^6^A-modified lncRNAs as candidates for functional studies and therapeutic targeting.

### Limitations of the study

First, the analysis was based on Calu-3 cells, a well-characterized but *in vitro* model of pulmonary epithelium. Therefore, the generalizability of these findings to other cell types, tissues, or *in vivo* contexts remains to be clarified. Second, although sequence logo and motif analyses revealed statistically significant remodeling of the DRACH motif landscape, these observations require experimental validation to confirm shifts in substrate specificity of the m^6^A methyltransferase complex during infection. Third, the rDNA-seq data was obtained from three independent infections, which were treated as technical replicates in the original study. While the cDNA-seq data were sequenced separately, the RNA samples were pooled for dRNA-seq due to multiplexing limitations in dRNA-seq kits (Oxford Nanopore Technologies). As a result, while dRNA-seq reflects the average behavior across infections, this pooling precludes replicate-aware statistical analyses, such as differential methylation testing with variance estimates. The focus of this study is to infer average changes in lncRNA expression and m^6^A patterns, and in that context, the pooled data remain valid for global trend analysis and hypothesis generation.

## Supplementary Material

ugaf034_Supplemental_Files

## Data Availability

The methylation workflow produced in this work is available on https://zenodo.org/records/16883738. All data and scripts in this study are available upon request to the corresponding author.
