# Supplementary material for: SARS-CoV-2 reshapes m6A methylation in long noncoding RNAs of human lung cells
Source: NAR Mol Med. 2025 Sep 30;2(4):ugaf034. doi: 10.1093/narmme/ugaf034 (PMC12628319; doi:10.1093/narmme/ugaf034)
Supplement: ugaf034_Supplemental_Files [file ugaf034_Supplemental_Files.zip › Supplementary Figures S1-S3.pdf]

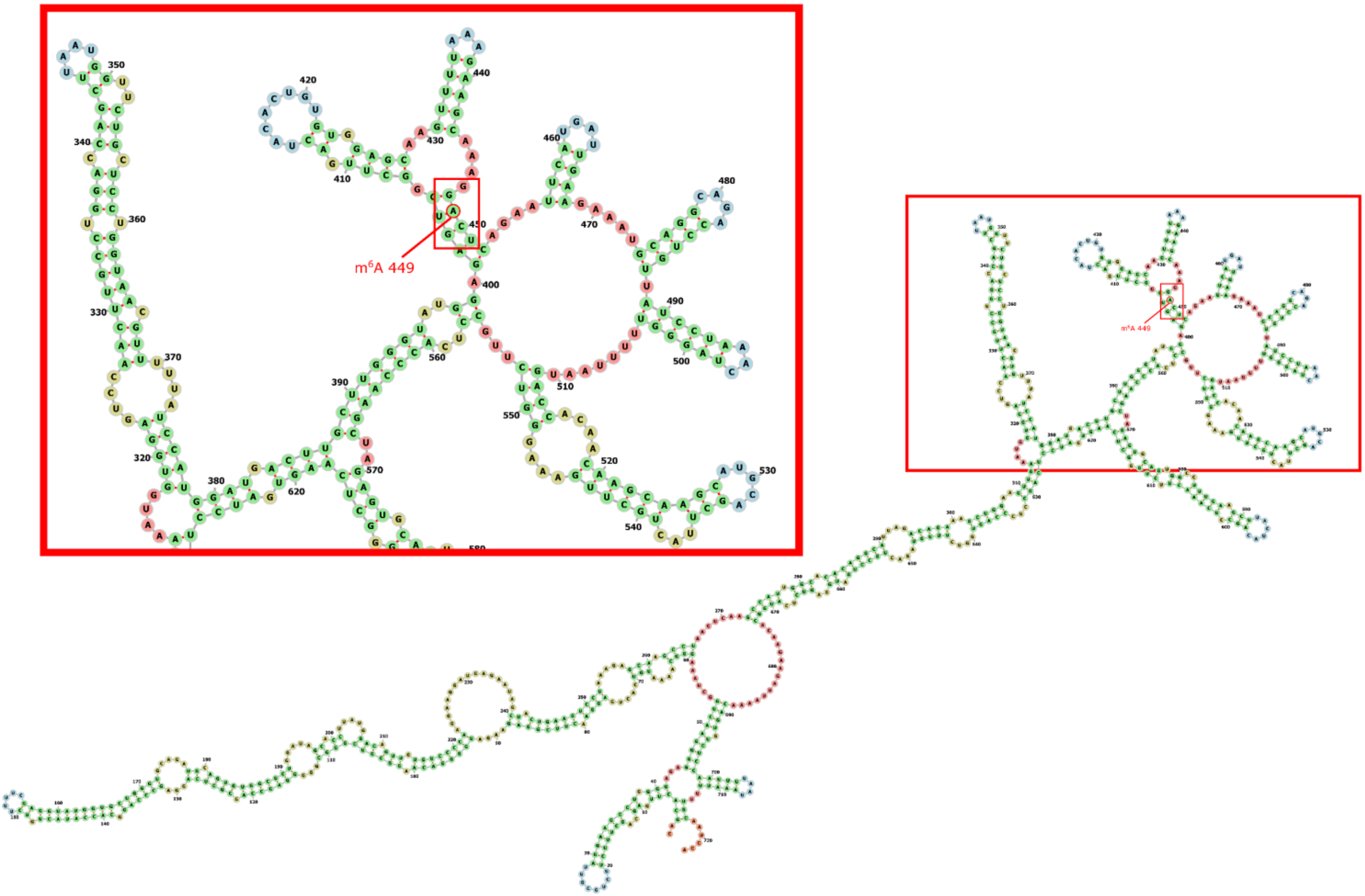

Supplementary Figure S1. Location of differentially methylated m^{6}A motifs in the secondary structure of GAS5 lncRNA. The structure was inferred by RNAfold and methylated adenosines detected by m6anet as in Figure 5 in the main text. The MFE of this structure is -237.50 kcal/mol. The red boxes detail the bona fide m^{6}A methylated domain. The DRACH motif GGACU is in the red box, the m6A in 449 is indicated by a red circle.

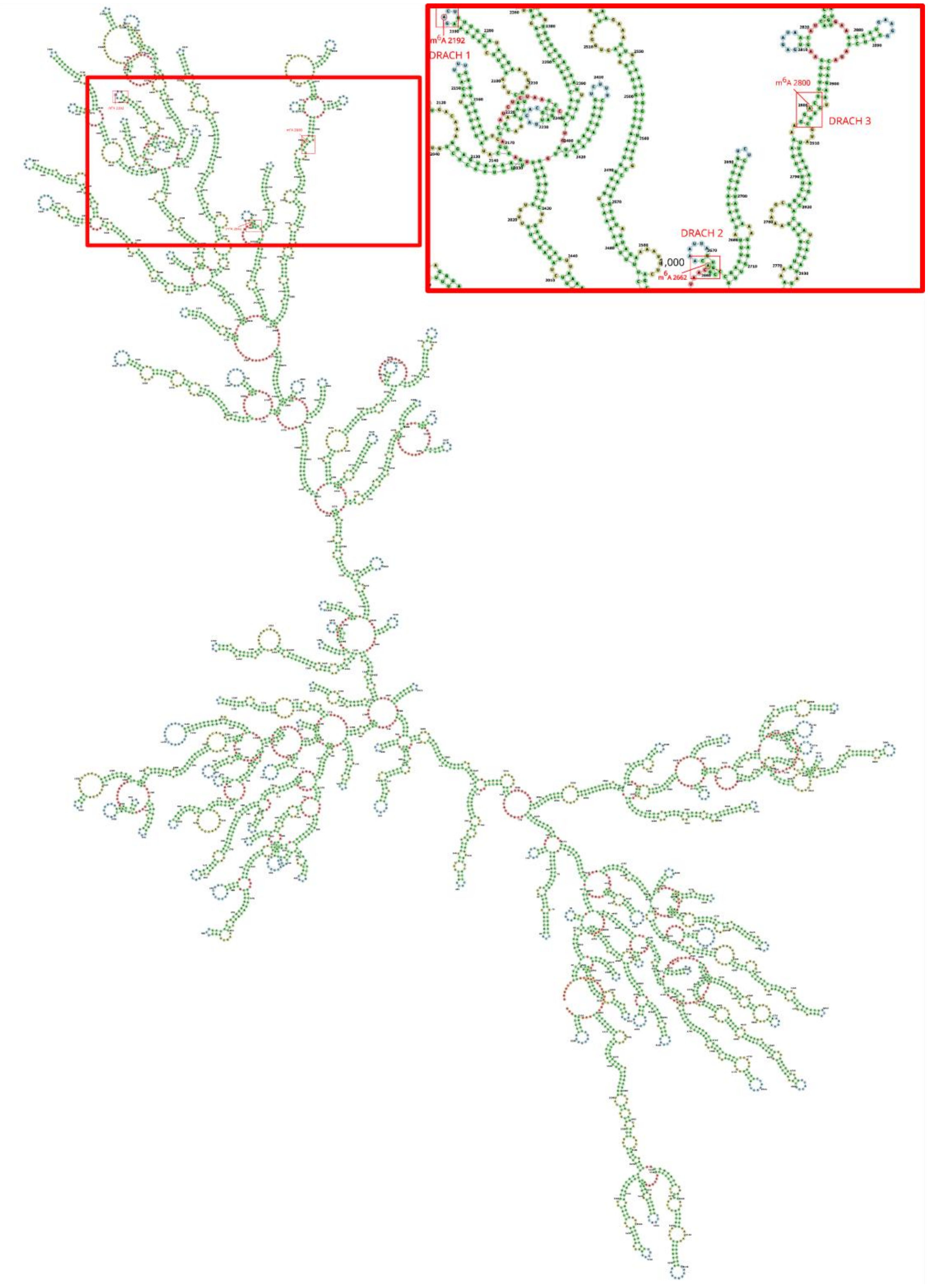

Supplementary Figure S2. Location of differentially methylated m^{6}A motifs in the secondary structure of NORAD lncRNA. The structure was inferred by RNAfold and methylated adenosines detected by m6anet as in Figure 5 in the main text. The MFE of this structure is -1657.50 kcal/mol. The red boxes detail the bona fide m^{6}A methylated domains. The DRACH motifs 1 (AGACU), 2 (GGACA) and 3 (GGACU) are in red boxes, m^{6}A in 2192, 2662 and 2800 are indicated by red circles.

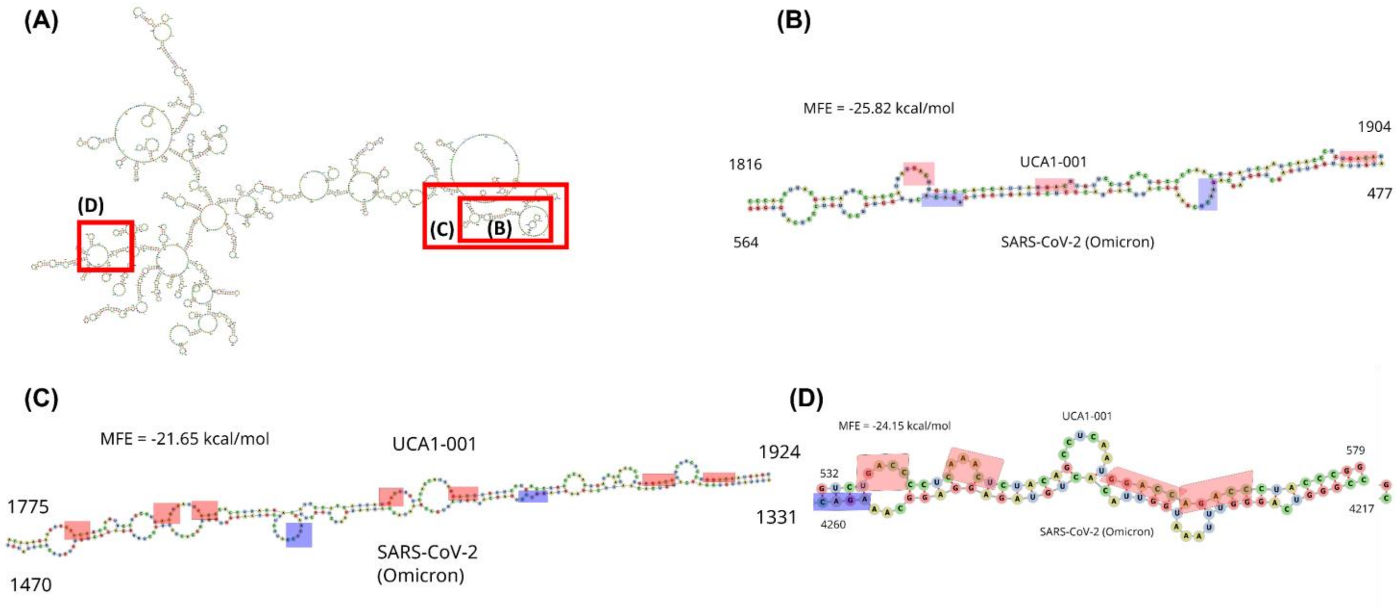

Supplementary Figure S3. Interactions of UCA1 lncRNA with SARS-CoV-2 Omicron RNA genome. (A) Location of interaction regions to SARS-CoV-2 genomic RNA (Omicron) in UCA1 lncRNA minimum free energy secondary structure. (B) UCA1 positions 1816 to 1904 are predicted to interact with SARS-CoV-2 RNA at positions 477 to 564 with Energy −25.82 kcal/mol. (C) Interactions at UCA1 positions 1775-1924 to SARS-CoV-2 (1331-1470) with Energy -21.65 kcal/mol and (D) UCA1 532-579 to SARS-CoV-2 4260-4217 with -24.15 kcal/mol. Light red boxes indicate DRACH motifs in lncRNA and blue boxes DRACH motifs in SARS-CoV-2 RNA. LncRNA-SARS-CoV-2 interactions were predicted using intaRNA program and confirmed by RNAhybrid program.
