# Supplementary material for: SARS-CoV-2 reshapes m6A methylation in long noncoding RNAs of human lung cells
Source: NAR Mol Med. 2025 Sep 30;2(4):ugaf034. doi: 10.1093/narmme/ugaf034 (PMC12628319; doi:10.1093/narmme/ugaf034)
Supplement: ugaf034_Supplemental_Files [file ugaf034_Supplemental_Files.zip › Supplementary Table S1.pdf]

Supplementary Table S1. The curated set of 100 lncRNAs initially analyzed.

Number	LncRNA	Related target	Regulation	Reads in Minimap2	Reference	
1	A2M-AS1 - Uninfected<br>A2M-AS1 -Infected<br>(ENSG00000245105)	miR-146b<br>PCBP3 (Poly(rC) Binding Protein 3)	A2M-AS1 has been shown to modulate inflammatory responses by regulating immune-related signaling pathways and interacting with key immune mediators such as PCBP3 and miR-146b.	0<br>0	Lin et al., (2023)	DOI 10.3389/fcell.2023.1229393
2	CRNDE - Uninfected<br>CRNDE - Infected<br>(ENSG00000245694)	miR-136, miR-217<br>NF-Kb, IL-6, STAT3, HMGA2	Elevated expression in PBMCs of patients with COVID-19, associated with inflammatory response.	44<br>28	Heydari et al., (2024)	doi.org/10.1186/s12985-024-02408-9
3	MEG9 - Uninfected<br>MEG9 - Infected<br>(ENSG00000223403)	S100A9, ICAM1, IL-6, CXCL6, MMP-7	MEG9 is a long non-coding RNA involved in promoting inflammation, liver fibrosis, and angiogenesis, acting as a key regulator in responses to genotoxic stress and inflammatory processes.	0<br>0	Meng et al., (2025)	doi.org/10.1016/j.jpedsurg.2024.07.018
4	LINC00487- Uninfected<br>LINC00487- Infected<br>(ENSG00000205837)	RSAD2 (Viperin), IL-27 / JAK/STAT1,<br>IFN-α	LINC00487 is a long non-coding RNA induced by IL-27 and IFN-α that promotes antiviral responses by upregulating RSAD2 expression through the JAK/STAT1 signaling pathway	5,875<br>7,309	Marin et al., (2025)	doi.org/10.1371/journal.pone.0314754
5	LINC00261 - Uninfected<br>LINC00261 - Infected<br>(ENSG00000259974)	IRF3	Activates antiviral response genes through interferon signaling and IRF3 activation	52<br>96	Ma et al., (2021)	doi: 10.3389/fonc.2021.653902
6	LINC00662 - Uninfected<br>LINC00662 - Infected<br>(ENSG00000261824)	miR-15a, miR-16 e miR-107, WNT3A	LINC00662 is a long non-coding RNA that promotes M2 macrophage polarization and tumor progression by sponging miR-15a, miR-16, and miR-107, thereby upregulating WNT3A and activating the Wnt/β-catenin signaling pathway.	2,059<br>2,522	Tian et al., (2020)	doi:10.1002/1878-0261.12606
7	BISPR - Uninfected<br>BISPR - Infected<br>(ENSG00000282851)	JAK-STAT, NF-κB	Regulation of BST2 (Tetherin); positive regulator of BST2 and part of the interferon-stimulated innate immune response.	13,594<br>15,728	Enguita et al., (2022)	doi: 10.7150/thno.73268
8	MROCKI - Uninfected<br>MROCKI- Infected<br>(ENSG00000227502) - LINC01268	MARCKS, PI3K/Akt, β-catenina	MROCKI is a long non-coding RNA located near the MARCKS gene and is proposed to regulate cytokine expression and responses. inflammatory signaling, potentially acting in cis to modulate immune	1,298<br>1,525	Tang et al., (2023)	DOI: 10.4251/wjgo.v15.i8.1366
9	C1RL-AS1 - Uninfected<br>C1RL-AS1 - Infected<br>(ENSG00000205885.9)	C1RL	Studies indicate that C1RL-AS1 is overexpressed in children with pneumonia caused by Influenza A virus, as well as in infected A549 cells.	2.562<br>3,069	Arman et al., (2023)<br>Turjya et al., (2020)	doi.org/10.1016/j.gene.2023.147232<br>DOI: 10.2217/fvl-2020-0188
10	SNHG1- Uninfected<br>SNHG1- Infected<br>(ENSG00000255717)	PI3K/AKT/mTOR, Wnt/β-catenina,<br>NF-κB, JAK/STAT<br>Sponge: miR-145, miR-195 e miR-326	SNHG1 functions as an oncogenic long non-coding RNA that promotes cell proliferation, inhibits apoptosis, and modulates inflammatory and signaling pathways such as PI3K/AKT and NF-κB.	12<br>31	Chen et al., (2020)	doi.org/10.3389/fonc.2020.552907
11	SNHG25- Uninfected<br>SNHG25- Infected<br>(ENSG00000266402)	miR-497-5p, SNORA50C	SNHG25 functions as an oncogenic long non-coding RNA that promotes tumor cell proliferation, migration, and survival by sponging tumor-suppressive microRNAs such as miR-497-5p and stabilizing oncogenic regulators like FASN and HDAC1	6<br>6	Arman et al., (2023)	doi.org/10.1016/j.gene.2023.147232
12	CASC2 - Uninfected<br>CASC2 - Infectedada<br>(ENSG00000177640.17)	NF-κB, MAPK/ERK, PI3K/AKT	Regulates the expression of cytokines such as IL-6, TNF-α, and IL-1β;<br>Regulation of inflammatory signaling pathways.	12,819<br>15,102	Ayoub et al., (2024)	doi.org/10.1186/s40246-024-00578-9
13	CDKN2B-AS1 - Uninfected<br>CDKN2B-AS1 - Infected<br>(ENSG00000240498)	NF-κB	NF-κB activation promotes the expression of pro-inflammatory genes.	14,154<br>16,471	Xiaoting et al., (2019), Hu et al., (2019)<br>Li et al., (2019), Badr et al., (2023)	10.1186/s40246-019-0240-4, doi.org/10.1016/j.biochi.2018.10.011<br>10.18632/aging.101863, 10.1186/s12879-023-08564-7
14	CEBPA-DT - Uninfected<br>CEBPA-DT - Infected<br>(ENSG00000267296)	hnRNPC<br>DDR2<br>β-catenina	CEBPA-DT promotes hepatocellular carcinoma metastasis by stabilizing DDR2 mRNA via hnRNPC interaction, transition. leading to β-catenin nuclear translocation and Snail1-mediated epithelial-mesenchymal	0<br>0	Cai et al., (2022)	doi.org/10.1186/s13046-022-02544-6
15	(CHROMR) - Uninfectede<br>(CHROMR) - Infected<br>(ENSG00000223960)	IL-6 and TNF-α	Promote the transcription of ISGs	8,287<br>9,603	van Solingena et al., (2022)	doi.org/10.1073/pnas.2210321119
16	CYTOR- LINC00152 - Uninf<br>CYTOR- LINC00152 - Infected<br>(ENSG00000222041)	CDK6, HOXC10, and SEMA4C	CYTOR is upregulated in severe COVID-19 cases and correlates with genes involved in neutrophil survival and inflammatory responses, suggesting its potential as a biomarker for disease severity.	12,970<br>15,270	Lim et al., (2024)<br>Aschenbrenner et al., (2021)	doi.org/10.1073/pnas.2404146121<br>doi.org/10.1186/s13073-020-00823-5
17	DANCR - Uninfected<br>DANCR - Infected<br>(ENSG00000226950)	NF-κB<br>IL-6 and TNF-α	Regulation of inflammation<br>Regulation of cell differentiation; promotion of cell proliferation and survival.	5,902<br>7,421	Laha et al., (2021)<br>Meydan et al., (2020)	doi.org/10.3390/ncrna7040074<br>doi: 10.3389/fimmu.2020.590870
18	EGOT - Uninfected<br>EGOT - Infected<br>(ENSG00000235947)	RSAD2 (Viperin), MX1 (Myxovirus resistance protein 1)<br>IFIT1 (Interferon-induced protein with tetratricopeptide repeats 1<br>OAS1 (2'-5'-Oligoadenylate Synthetase 1), ISG15	It acts as an interferon-stimulated lncRNA that, paradoxically, inhibits the expression of other ISGs, functioning as a negative regulator of the IFN-dependent antiviral axis.	3<br>2	Carnero et al., (2016)	DOI 10.15252/embr.201541763
19	FAM106A - Uninfected<br>FAM106A - Infected<br>(ENSG00000273018)	JAK-STAT, Wnt/β-catenina and MAPK	This downregulation could lead to overexpression of FAM106A target microRNAs, resulting in pro-inflammatory and pro-fibrotic events.	88<br>99	Talotta et al., (2021)	doi.org/10.1016/j.bbadis.2021.166291
20	RORA-AS1 - Uninfected<br>RORA-AS1 - Infected<br>(ENSG00000245534)	RORA	RORA-AS1 is an antisense long non-coding RNA that potentially regulates the expression of the RORA gene, influencing pathways related to inflammation, lipid metabolism, and circadian rhythm.	18,077<br>20,756	Liu et al., (2023)<br>Cai et al., (2020)	doi.org/10.1158/0008-5472.CAN-23-3942<br>doi.org/10.3892/mmr.2020.11558
21	FENDRR - Uninfected<br>FENDRR - Infected<br>(ENSG00000268388)	NF-κB, MAPKs	Regulation of inflammation<br>FENDRR acts as an epigenetic modulator, interacting with complexes such as PRC2 (Polycomb Repressive Complex 2) and TrxG/MLL, promoting the methylation of target gene promoters and, consequently, reducing their expression	449<br>679	Saha et al., (2021)	doi.org/10.3390/ncrna7040074
22	FIRRE - Uninfected<br>FIRRE - Infected<br>Also known as (LINC01200)<br>(ENSG00000213468)	VCAM1, IL12B (IL-12p40), MX1, BECN1<br>Cyclin D1 (CCND1) and CCND3, LYL1, CTBP1	FIRRE may influence the expression of genes involved in viral entry or immune responses.<br>May interact with epigenetic modifiers to alter the expression of host genes critical for viral replication or immune evasion.	19,515<br>22,398	Lu et al., (2017)	doi/10.4049/jimmunol.1700091
23	FOSL2-AS1 - Uninfected<br>FOSL2-AS1 - Infected<br>(ENSG00000229951)	FOSL2	FOSL2-AS1 may promote the activation and differentiation of T cells, and potentially enhance their functional responses.	7,033<br>8,155	Lin et al., (2023)	DOI 10.3389/fcell.2023.1229393
24	GAS5 - Uninfected<br>GAS5 - Infected<br>(ENSG00000234741)	TNF-α/IL-10	Suppresses NF-κB activation, reducing the release of pro-inflammatory cytokines such as TNF-α, IL-6, and IL-1β.	2,247<br>2,655	Ayeldeen et al., (2024), Curci et al., (2024)<br>Li et al., (2017), Xiao and Wang (2023)	DOI 10.2174/0929867330666230515144133, DOI 10.3389/fphar.2024.1448136<br>DOI 10.1007/s10620-017-4831-4, 10.2174/1381612829666230517102205
25	GAS6-AS1- Uninfected<br>GAS6-AS1- Infected<br>(ENSG00000233695)	TRIM14	GAS6-AS1 acts as a ceRNA, sequestering microRNAs (such as miR-370-3p) that would normally repress TRIM14 expression	5,430<br>6,854	Turjya et al., (2020)	DOI: 10.2217/fvl-2020-0188
26	HAND2-AS1 - Uninfected<br>HAND2-AS1 - Infected<br>(ENSG00000237125)	LEPR, KLF9, RARB	Acts as a suppressor of pro-inflammatory pathways, and its expression negatively regulates the production of IL-6	12,643<br>14,692	Arman et al., (2023)	doi.org/10.1016/j.gene.2023.147232
27	HIF1A-AS1 - Uninfected<br>HIF1A-AS1 - Infected<br>(ENSG00000258777)	MAPK	T cell activation and differentiation	6,927<br>8,538	Li et al., (2015)	doi: 10.1093/bib/bbu048
28	HOTAIR - Uninfected<br>HOTAIR - Infected<br>(ENSG00000228630)	NF-κB (RelA/p65), IκBα, IL-6, Inos	HOTAIR promotes IL-6 expression by activating the NF-κB pathway, facilitating the degradation of the inhibitor IκBα, which allows the translocati of NF-κB to the nucleus, where it activates inflammatory genes such as IL6 and iNOS.	0<br>0	Khanaliha et al., (2024)	DOI: 10.1002/hsr2.1861
29	HOTAIRM1 - Uninfected<br>HOTAIRM1 - Infected<br>(ENSG00000233429)	HOXA1, miR-125b, IL-6	HOTAIRM1 is a myeloid-specific long non-coding RNA that regulates cell differentiation and inflammatory responses by modulating HOXA gene expression and acting as a competing endogenous RNA	0<br>2	Moazzam-Jazi et al., (2021)	DOI: 10.1111/jcmm.16596
30	HULC - Uninfected<br>HULC - Infected<br>(ENSG00000285219)	IL-6, CXCR4, miR-372/373 e miR-9	HULC is a long non-coding RNA that contributes to inflammatory responses by sponging miR-9, thereby enhancing IL-6 expression and correlating with increased disease severity in COVID-19 patients	0<br>0	Esawy et al.,(2021)	DOI: 10.3934/molsci.2022005
31	(IFNG-AS1) - Uninfected<br>(IFNG-AS1) - Infected<br>(ENSG00000255733)	IFNG (Interferon-gama), IL6, IL1B	IFNG-AS1 acts as a positive regulator of IFN-γ expression in Th1 cells, promoting the cellular immune response against intracellular pathogens.<br>Modulates IFN-γ expression and enhances the antiviral response.	6,927<br>8,538	Laha et al., (2021)	doi.org/10.1016/j.heliyon.2021.e06395
32	IL6-AS1 - Uninfected<br>IL6-AS1 - Infected<br>(ENSG00000179428)	miR-149-5p, IL-6	Due to its antisense location relative to the IL6 gene, IL6-AS1 may influence IL6 expression by mechanisms of transcriptional interference or epigenetic modulation.	2<br>45	Zhong et al., (2022)	DOI 10.3389/fmolb.2022.975322
33	INHBA-AS1 - Uninfected<br>INHBA-AS1 - Infected<br>(ENSG00000224116)	INHBA, TGF-β	As an antisense RNA, INHBA-AS1 may regulate INHBA expression by transcriptional interference or epigenetic modulation mechanisms.	10,475<br>12,205	Turjya et al., (2020)	DOI: 10.2217/fvl-2020-0188
34	KCNQ1OT1 - Uninfecteded<br>KCNQ1OT1 -Infected<br>(ENSG00000269821)	NF-κB<br>miR-370-3p/FOXM1	Role in cell survival and regulation of gene expression and Interact with upregulated TLR2	7,949<br>9,440	Dai et al., (2018); Tian et al., (2018)	doi.org/10.1080/03008207.2018.1439480, DOI 10.1038/s41419-018-0925-y
35	LEF1-AS-1 - Uninfected<br>LEF1-AS-1 - Infected<br>(ENSG00000232021)	miR-222-5p, RAMP3, PIK3R2/PI3K/AKT	T cell activation and differentiation (Th17 cell differentiation)	3,417<br>4,024	Cheng et al. (2020)<br>Zheng et al., (2021)	doi.org/10.1038/s41419-020-02823-0<br>doi: 10.3389/fcell.2021.750084
36	LINC00173 - Uninfected<br>LINC00173 - Infected<br>(ENSG00000196668)	miR-1275, PROCA1/ZFP36L2/BCL2<br>SNAIL/FHIT	LINC00173 may interact with transcription factors such as SNAIL, influencing the expression of tumor suppressor genes such as FHIT. a<br>This interaction is relevant in contexts such as lung adenocarcinom	5,161<br>5,999	Suzuki et al., (2023)	doi.org/10.3390/ijms242317011
37	LINC00273 - Uninfected<br>LINC00273 - Infectedada<br>(ENSG00000256642)	NF-κB	Regulation of inflammation<br>LINC00273 pode influenciar a secreção de citocinas pró-inflamatórias, como IL-6 e TNF-α, dependendo do contexto celular	16<br>38	Chattopadhyay et al., (2022)	10.3389/fimmu.2022.1035111
38	LINC00278 - Uninfected<br>LINC00278 - Infected<br>(ENSG00000231535)	NF-κB	Biomarker during SARS-CoV-2 infection	9,734<br>11,510	Cheng et al., (2021)<br>Talotta et al., (2021)	DOI: 10.1111/jcmm.16444<br>doi.org/10.1016/j.bbadis.2021.166291
39	LINC00305 - Uninfected<br>LINC00305 - Infected<br>(ENSG00000179676)	NF-κB	LINC00305 enhances inflammation by activating the NF-κB signaling pathway in monocytes, contributing to the upregulation of pro-inflammatory genes	10,722<br>12,563	Morenikeji et al.(2021)<br>Bergara-Muguruza et al. (2023)	doi: 10.3389/fbioe.2020.582953<br>DOI: 10.20517/itgg.2023.14
40	LINC00473 - Uninfected<br>LINC00473 - Infected<br>(ENSG00000112541)	NF-κB<br>P65, STAT3 e CREB	Regulation of pro-inflammatory cytokines, Interaction with signaling pathways.	7,681<br>9,170	Saha et al., (2021)<br>Laha et al., (2021)	doi.org/10.3390/ncrna7040074<br>doi.org/10.1016/j.heliyon.2021.e06395
41	LINC00504 - Uninfected<br>LINC00504 - Infected<br>(ENSG00000248360)	STAT1	It acts as a molecular sponge for the microRNA miR-140-5p, releasing the expression of the VEGFA gene.	16,594<br>19,072	Turjya et al., (2020)	DOI: 10.2217/fvl-2020-0188
42	LINC00511 - Uninfected<br>LINC00511 - Infected<br>(ENSG00000227036)	NF-κB	Acts as a competing endogenous RNA (ceRNA), interacting with specific microRNAs (miRNAs) and modulating target gene expression.<br>May modulate miR-29b-3p and miR-150, which are known to regulate the inflammatory response.	14,194<br>16,440	Taheri et al., (2021)	doi.org/10.1186/s12879-021-06248-8
43	LINC00963 - Uninfected<br>LINC00963 - Infected<br>(ENSG00000204054)	miR-1281/TRIM65, miR-10b/FGF13<br>miR-324-3p/ACK1	LINC00963 may modulate the expression of genes involved in viral replication or immune evasion	14,509<br>16,704	Li et al., (2025)	doi.org/10.1186/s13018-025-05744-w
44	LINC01018 - Uninfected<br>LINC01018 - Infected<br>(ENSG00000250056)	miR-942-5p / KNG1<br>miR-499a-5p / PDCD4<br>miR-182-5p / FOXO1, miR-182-5p	LINC01018 may influence the expression of genes related to the inflammatory response, possibly affecting the production of pro-inflammatory cytokines such as IL-6 and TNF-α.	24<br>24	Wang et al., (2019)	doi:10.1152/ajpgi.00005.2019
45	LINC01133 - Uninfected<br>LINC01133 - Infected<br>(ENSG00000224259)	miR-30b-5p / Rab3D, miR-106a-3p / APC<br>EZH2 / AXIN2	LINC01133 is involved in the regulation of signaling pathways such as Wnt/β-catenin, influencing processes such as cell proliferation, migration and invasion.	3.279<br>3,815	Jiang et al., (2022)	doi: 10.3389/fonc.2022.908162
46	LINC01355 - Uninfected<br>LINC01355 - Infected<br>(ENSG00000261326)	FOXO3, CCND1 (Ciclina D1), Notch1, JAG1, HES1	LINC01355 functions as a context-dependent regulator, acting as a tumor suppressor by stabilizing FOXO3 and repressing CCND1 in breast cancer, while promoting tumor progression through activation of the Notch signaling pathway in oral squamous cell carcinoma	383<br>495	Zou et al., (2021)	doi.org/10.1155/2021/1830790
47	LINC01410 - Uninfected<br>LINC01410 - Infected<br>(ENSG00000238113)	miR-30b-3p, miR-3128<br>miR-506-3p, STAT5	LINC01410 functions as an oncogenic lncRNA that promotes tumor progression by activating key signaling pathways such as ErbB and Notch, primarily through sponging tumor-suppressive microRNAs like miR-506-3p and miR-3128.	0<br>0	Lu et al., (2021)	doi: 10.3389/fonc.2021.659123
48	LINC01426 - Uninfected<br>LINC01426 - Infected<br>(ENSG00000234380)	miR-345-3p / VAMP8, miR-519d-5p / ETS1	LINC01426 functions as an oncogenic lncRNA that promotes tumor progression by sponging tumor-suppressive microRNAs such as miR-345-3p and miR-519d-5p, leading to the upregulation of pro-tumorigenic targets like VAMP8 and ETS1 in glioblastoma and lung adenocarcinoma, respectively	12,508<br>14,492	Cao et al., (2020)	doi.org/10.1186/s12935-020-01416-3
49	LINC01503 - Uninfected<br>LINC01503 - Infected<br>(ENSG00000233901)	miR-615-3p / CCND1, miR-4492 / FOXK1<br>miR-342-3p / LASP1, SFPQ / FOSL1	LINC01503 acts as an oncogenic lncRNA that promotes tumor progression by sponging tumor-suppressive microRNAs and recruiting epigenetic regulators, thereby enhancing the expression of oncogenes across multiple cancer types	0<br>0	Shua et al., (2024)	doi.org/10.1007/s10238-024-01383-3
50	LINC01505 - Uninfected<br>LINC01505 - Infected<br>(ENSG00000234323)	PER1 (Period Circadian Regulator 1)	SARS-CoV-2 Spike protein genome or mRNA may interact with lncRNAs to modulate viral replication.	14,143<br>16,299	Lin et al., (2023)	DOI 10.3389/fcell.2023.1229393
51	LINC01572 - Uninfected<br>LINC01572 - Infected<br>(ENSG00000261008)	miR-195-5p, PFKFB4, PI3K/AKT	LINC01572 promotes hepatocellular carcinoma progression by sponging miR-195-5p, leading to upregulation of PFKFB4 and activation of the PI3K/AKT signaling pathway, thereby enhancing glycolysis and tumor cell proliferation.	0<br>0	Lai et al., (2021)	doi: 10.3389/fcell.2021.783088
52	LINC01614 - Uninfected<br>LINC01614 - Infected<br>(ENSG00000230838)	miR-138-5p, FOXC1, miR-4775, ANXA2 / p65<br>SLC38A2 / SLC7A5	LINC01614 promotes tumor progression by sponging miR-138-5p and miR-4775 or activating the NF-κB signaling pathway via interaction with ANXA2/p65, thereby enhancing FOXC1 expression, drug resistance, and glutamine uptake in cancers such as OSCC, ESCC, and lung adenocarcinoma	0<br>0	Liu et al., (2022)	doi.org/10.1186/s13045-022-01359-4
53	LINC01619 - Uninfected<br>LINC01619 - Infected<br>(ENSG00000257242)	NF-κB	Biomarker during SARS-CoV-2 infection	14,926<br>17,445	Cheng et al., (2021)<br>Zhong et al.,(2022)	DOI: 10.1111/jcmm.16444<br>DOI 10.3389/fmolb.2022.975322
54	LINC01988 - Uninfected<br>LINC01988 - Infected<br>(ENSG00000283036)	NF-κB	Biomarker during SARS-CoV-2 infection	5,130<br>6,102	Cheng et al., (2021)	DOI: 10.1111/jcmm.16444
55	LINC02015 - Uninfected<br>lINC02015 - Infected	PI3K/AKT e MAPK	Role in cell survival and regulation of gene expression	13,943<br>16,208	Turjya et al., (2020)	DOI: 10.2217/fvl-2020-0188

	(ENSG00000231574)					
56	LINC02384 - Uninfected<br>LINC02384- Infected<br>(ENSG00000251301)	IFN	Limit innate immune	2,109<br>2,459	Yang et al., (2021)	doi: 10.3389/fimmu.2021.700184
57	LINC02390 - Uninfected<br>LINC02390 - Infected<br>(ENSG0000025658)	NF-κB	Biomarker during SARS-CoV-2 infection	92<br>124	Cheng et al., (2021)	DOI: 10.1111/jcmm.16444
58	LINC00937 - Uninfected<br>LINC00937 - Infected<br>(ENSG00000226091)	IL10RA	Modulation of Apoptosis	13,386<br>14,825	Hu et al., (2019)	doi:10.3390/ijms20246207
59	LINC00673 - Uninfected<br>LINC00673 - Infected<br>(ENSG00000227036)	NF-κB or STAT3	Regulates cell signaling pathways and gene expression.	0<br>0	Zhu et al., (2021)	doi: 10.7150/ijms.48134
60	LINC02574 - Uninfected<br>LINC02574 - Infected<br>(ENSG00000233975)	NF-κB	Regulation of inflammation, Acts by inhibiting viral replication through the positive regulation of the<br>innate immune response.	1,104<br>1,073	Zhang et al., (2023)	doi.org/10.3390/ijms24087248
61	LNCATV - Uninfected<br>LNCATV - Infected<br>(ENSG00000238005)	TBK1 (TANK-binding kinase 1)<br>IRF3 (Interferon Regulatory Factor 3)<br>RIG-I (DDX58), MDA5	Inhibition of RLR (RIG-I-like receptors) Signaling<br>Interference with TBK1 and IRF3	2,174<br>2,717	van Solingena et al., (2022)<br>Enguita et al., (2022)	doi.org/10.1073/pnas.2210321119<br>doi: 10.7150/thno.73268
62	LUCAT1 - Uninfected<br>LUCAT1 - Infected<br>(ENSG00000248323)	NF-κB	Regulation of inflammation<br>Induced expression of proinflammatory markers CXCL2 and CXCL8	8,521<br>9,912	Aznaourova et al. (2022)	doi.org/10.1073/pnas.2120680119
63	MALAT1 - Uninfected<br>MALAT1 - Infected<br>(ENSG00000251562)	NF-κB	Induced TNF-α、IL-6、IL-1 and CCL2<br>Regulation of inflammation	18<br>19	Enguita et al. (2022), Dai et al.(2018), Rodrigues et ., (2021)<br>Tian et al., (2018), Hao et al., (2020) and Xu et. (2024)	doi: 10.7150/thno.73268, doi.org/10.1080/03008207.2018.1439480, DOI:10.1111/omi.12351<br>DOI 10.1038/s41419-018-0925-y, doi.org/10.1016/j.virusres.2020.197907, doi.org/10.1016/j.ncrna.2024.01.015
64	MEG3 - Uninfected<br>MEG3 - Infected<br>(ENSG00000214548)	NF-κB	Regulation of inflammation, Downregulates inflammation, Inhibits NF-κB, regulates epigenetics	6,599<br>7,392	Turjya et al., (2020)	DOI: 10.2217/fvl-2020-0188
65	MIR155HG - Uninfected<br>MIR155HG - Infected<br>(ENSG00000234883)	NF-Kb	miR-155 Precursor<br>Regulation of Innate and Adaptive Immune Responses<br>Amplification of Inflammatory Signaling	1,348<br>1,804	Rai et al., (2022)<br>Enguita et al., (2022)	doi: 10.1128/mbio.02510-22<br>doi: 10.7150/thno.73268
66	MIR222HG - Uninfected<br>MIR222HG - Infected<br>(ENSG00000270069)	NF-κB	Biomarker during SARS-CoV-2 infection	5,231<br>6,433	Sun et al., (2018)<br>Wen et al., (2023)	DOI 10.1038/s41389-018-0039-5<br>doi: 10.3389/fimmu.2023.1168920.
67	MIR3142HG - Uninfected<br>MIR3142HG - Infected<br>(ENSG00000253522)	PI3K/AKT e MAPK	Role in cell survival and regulation of gene expression	7,671<br>8,745	Hadjicharalambous et al., (2018)	doi: 10.3389/fimmu.2018.02906
68	NCBP2-AS1- Uninfected<br>NCBP2-AS1-Infected<br>(ENSG00000225578)	NF-κB	Biomarker during SARS-CoV-2 infection	5,260<br>6,319	Li et al., (2025)<br>Badr et al.,(2023)	doi: 10.7717/peerj.19050<br>doi.org/10.1186/s12879-023-08564-7
69	NEAT1 - Uninfected<br>NEAT1 - Infected<br>(ENSG00000245532)	NF-κB	Regulation of inflammation<br>Induced TNF-α, IL-6, and IL-1, NLRP3, as well as CCl2, Ccl3, and CXCL10	3,655<br>4,532	Dai et al., (2018); Moazzam-Jazi et al., (2021), Vishnubalaji et a<br>Tian et al., (2018); Meydan et al., (2020), Rodrigues et ., (2021)	doi.org/10.1080/03008207.2018.1439480, DOI: 10.1111/jcmm.16596, doi:10.3390/genes11070760<br>DOI 10.1038/s41419-018-0925-y; doi: 10.3389/fimmu.2020.590870, DOI:10.1111/omi.12351
70	NKILAUninfected<br>NKILA - Infected<br>(ENSG00000278709)	NF-κB	Regulation of inflammation<br>Inadequate regulation of NKILA may lead to uninfected activation of NF-κB, exacerbating the "cytokine storm".	77<br>64	Ye et al., (2022)	doi.org/10.3390/cancers14092101
71	NIFK-AS1 - Uninfected<br>NIFK-AS1 - Infected<br>(ENSG00000236859)	SRSF10 (splicing factor), METTL3 (m^{6}A writer), miR-146a<br>Notch1,miR-647	Suppressed Innate Immunity<br>Negatively Regulates M2 polarization and Macrophage activation	21.299<br>23,995	Chattopadhyay et al., (2022)	DOI 10.3389/fimmu.2022.1035111
72	NORAD - Uninfected<br>NORAD - Infected<br>(ENSG00000260032)	NF-κB	Induced IL-6、IL-10、CSF3、CXCL10、TNF-α	3,587<br>4,467	Morenikeji et al. (2021)<br>Yang et al., (2021)	doi: 10.3389/fbioe.2020.582953<br>doi.org/10.3389/fimmu.2021.700184
73	NRAV - Uninfected<br>NRAV - Infected<br>(ENSG00000248008)	NF-κB	ISG denial: NRAV represses the expression of interferon-stimulated genes, which are crucial for the innate antiviral response.	7,912<br>9,277	Li et al., (2022)	doi.org/10.1007/s11033-022-07268-6
74	NRON - Uninfected<br>NRON - Infected<br>(ENSG00000253079)	NF-κB	Regulação da atividade do fator de transcrição NFAT (Nuclear Factor of Activated T-cells)	427<br>555	Imam et al., (2015)	DOI: 10.1038/srep08639
75	NRIR - Uninfected<br>NRIR - Infected<br>(ENSG00000225964)	CMPK2, STAT1, STAT2, IRF3, IRF7	In hepatitis C virus (HCV)-infected hepatocytes, NRIR is induced by IFN-α and acts as a negative regulator of ISG expression,<br>facilitating viral replication.	4,678<br>5,382	Sefatjoo et al., (2024)<br>Lin et al., (2023)	doi.org/10.1016/j.cyto.2023.156495<br>doi:10.3389/fcell.2023.1229393
76	PACERR - Uninfected<br>PACERR - Infected<br>(ENSG00000273129)	miR-671-3p, KLF12, IGF2BP2, CTCF and EP300	PACERR is a long non-coding RNA that enhances COX-2 (PTGS2) transcription by interacting with the NF-κB p50 subunit,<br>facilitating pro-inflammatory gene expression.	0<br>1	Liu et al.,(2022)	doi.org/10.1186/s13045-022-01272-w
77	PIRAT - Uninfected<br>PIRAT - Infected<br>(ENSG00000237803)	NF-κB	Regulation of inflammation, Reduce alarmin production	9,867<br>11,686	Aznaourova et al.(2022)	doi.org/10.1073/pnas.2120680119
78	PVT-1Uninfected<br>PVT-1 - Infected<br>(ENSG00000249859)	MYC, miR-152,miR-145, miR-1207-3p, miR-1301-3p	PVT1 interacts with epigenetic proteins such as EZH2 and components of the PRC2 complex, regulating the expression of target genes/ PVT1 act<br>as a microRNA "sponge; m6A methylation may modulate the stability and function of PVT1,influencing its interaction with proteins and other RNA	19,039<br>21,848	Moazzam- Jazi et al., (2021)	DOI: 10.1111/jcmm.16596
79	PYCARD - Uninfected<br>PYCARD- Infected<br>(ENSG00000103490)	Caspase-1 (CASP1), IL-1β, L-18, Gasdermin D, NLRP3, AIM2,<br>NLRC4, NLRP1, TLR4, TLR2, TLR9	PYCARD expression is correlated with immunological subtypes in several human cancers, decreasing its role in modulating the<br>tumor microenvironment.	90<br>86	Miao et al., (2019)	doi.org/10.1371/journal.pgen.1008144
80	Pycard-AS1 - Uninfected<br>Pycard-AS1 - Infected<br>(ENSG00000261359)	PYCARD (ASC), Caspase-1 (CASP1), IL-1β, IL-18<br>GSDMD (Gasdermin D), Inflamasoma NLRP3/AIM2	By suppressing PYCARD expression, PYCARD-AS1 decreases inflammasome activation and caspase-1-mediated apoptosis,<br>affecting the inflammatory response and programmed cell death.	83<br>76	Miao et al., (2019)	doi.org/10.1371/journal.pgen.1008144
81	RAD51-AS1 - Uninfected<br>RAD51-AS1 - Infected<br>(ENSG00000245849)	NF-κB	Regulation of inflammation<br>Induced IL-6、TNF-α、CCL2	54<br>41	Morenikeji et al.(2021)	doi: 10.3389/fbioe.2020.582953
82	RCC2-AS1 - Uninfected<br>RCC2-AS1 - Infected<br>(ENSG00000227751)	RCC2	Biomarker during SARS-CoV-2 infection	61<br>97	(Vishnubalaji et al., 2020)	doi:10.3390/genes11070760
83	RDUR - Uninfected<br>RDUR - Infected<br>(ENSG00000214407)	NF-κB<br>IRF3 e IRF7	They modulate the innate immune response mediated by the RIG-I<br>(Retinoic Acid-Inducible Gene I) receptor; RIG-I–dependent antiviral response regulator RNA	11,922<br>13,965	Chen et al., (2021)	doi.org/10.3389/fimmu.2021.672165
84	ROR1-AS1 - Uninfected<br>ROR1-AS1 - Infected<br>(ENSG00000223949)	NF-κB	ROR1-AS1 is implicated in the activation of the NF-κB signaling pathway, a central<br>regulator of inflammatory responses	5,991<br>7,058	Chattopadhyay et al. (2022)	10.3389/fimmu.2022.1035111
85	SNHG19 - Uninfected<br>SNHG19 - Infected<br>(ENSG00000260260)	miR-137, miR-299-5p<br>miR-299-5p, miR-137	SNHG19 acts as a sponge for specific miRNAs, such as miR-137 and miR-299-5p, preventing them from repressing their gene targets.	2<br>0	(Vishnubalaji et al., 2020)	doi:10.3390/genes11070760
86	SNHG6 - Uninfected<br>SNHG6 - Infected<br>(ENSG00000245910)	miR-101-3p, miR-186-5p	It may play a role in regulating the cytokine storm, one of the key mechanisms underlying disease severity.<br>The decreased expression of SNHG6 in COVID-19 suggests that it may be involved in modulating the inflammatory response during infection	2,189<br>2.381	Taheri et al., (2021)	doi.org/10.1186/s12879-021-06248-8
87	SNHG7 - Uninfected<br>SNHG7 - Infectedad<br>(ENSG00000233016)	miRNAs, NF-κB, IL-6, TNF-α<br>Bcl-2, Bax, Caspase-3<br>PI3K/AKT/mTOR, JAK/STAT e MAPK	SNHG7 modulates inflammatory cytokines, Promotes the activation of inflammatory pathways, such as NF-κB and JAK/STAT<br>May interfere with the production of type I interferons (IFN-α and IFN-β),modulating the innate antiviral response against SARS-CoV-2.<br>MiRNA sponge, Regulation of Viral Replication	5,525<br>6,452	(Vishnubalaji et al., 2020)	doi:10.3390/genes11070760
88	SNHG8 Uninfected<br>SNHG8 Infected<br>(ENSG00000269893)	NF-κB, PI3K/AKT<br>miRNAs-chave<br>Bcl-2, Cyclin D1	Role in cell survival and regulation of gene expression<br>Inhibition of apoptosis, Activation of the PI3K/AKT pathway, Promotion of cellular<br>Evasion of cellular stress	29<br>19	(Vishnubalaji et al., 2020)	doi:10.3390/genes11070760
89	SNHG14 - Uninfected<br>SNHG14 - Infected<br>(ENSG00000224078)	miR-223-3p/Foxo3a, miR-136-5p/ROCK1<br>miR-5590-3p/ZEB1/PD-L1	Regulates cell proliferation, apoptosis, and immune responses.<br>SNHG14 may modulate the expression of genes involved in viral replication or immune evasion.	0<br>0	Hong et al., (2021)	doi.org/10.1155/2021/7890288
90	THRIL - Uninfected<br>THRIL - Infected<br>(ENSG00000280634)	NF-κB	Induces TNF-α; TNF- and HNRNPL-related immunoregulatory activity; amplifies the<br>innate immune response	3.787<br>4,323	Rahni et al., (2023)	doi.org/10.1016/j.virusres.2023.199214
91	TUG1 - Uninfected<br>TUG1 - Infectedada<br>(ENSG00000253352)	NF-κB	Regulates inflammation by sponging this miRNA, which<br>may influence the activation of factors such as NF-κB	35<br>47	Tayel et al., (2022)<br>Wang et al. (2022)	doi: 10.7150/ijbs.72318 ,<br>doi.org/10.1186/s42494-022-00080-6
92	UCA1 - Uninfected<br>UCA1 - Infected<br>(ENSG00000214049)	NF-κB	Negatively regulates CD8 T cell activation through PD-L1<br>Positively regulates TNF-a, IL6, IL1b, Suppressed Inflammatory Response	137<br>129	Chattopadhyay et al., (2022)<br>Yang et al., (2024)	DOI 10.3389/fimmu.2022.1035111<br>doi.org/10.1038/s41417-024-00734-2
93	UGDH-AS1 - Uninfected<br>UGDH-AS1 - Infected<br>(ENSG00000249348)	UGDH<br>miRNAs como miR-129-5p e miR-1	Role in cell survival and regulation of<br>gene expression	19,215<br>22,049	Chattopadhyay et al., (2022)	DOI 10.3389/fimmu.2022.1035111
94	USP30-AS1 - Uninfected<br>USP30-AS1 - Infected<br>(ENSG00000256262)	USP30	Regulation of inflammation	2<br>5	Enguita et al., (2022)	doi: 10.7150/thno.73268
95	XIST - Uninfected<br>XIST - Infected<br>(ENSG00000229807)	PRC2 (Polycomb Repressive Complex 2)<br>SPEN (Split Ends), MacroH2A<br>SMRT/HDAC, CBX Proteins	Biomarker during SARS-CoV-2 infection	6,726<br>7.876	Vishnubalaji et al., (2020)	doi:10.3390/genes11070760
96	WAKMAR2 - Uninfected<br>WAKMAR2 - Infected<br>(ENSG00000237499)	miR-4478 / E2F1 / p53<br>c-Fos / CLDN1	WAKMAR2 acts as a regulatory RNA in inflammation and modulation of the immune response.	11,069<br>13,070	Mukherjee et al.,(2021)<br>Wang et al., (2021)	doi.org/10.3390/v13030402<br>doi.org/10.1038/s41598-021-94784-3
97	SGMS1-AS1 - Uninfected<br>SGMS1-AS1 - Infected<br>(ENSG00000226200)	miR-106a-5p<br>MYLIP (Myosin Regulatory Light Chain Interacting Protein)	Predicted to act upstream of or within positive regulation of myelination and regulation of gene expression.	1,133<br>1,215	Arman et al., (2023)	doi.org/10.1016/j.gene.2023.147232
98	PRNCR1 - Uninfected<br>PRNCR1 - Infected<br>(ENSG00000282961)	miR-326 / FSCN1, miR-182-5p / EZH1<br>miR-653-5p / ELF2, miR-126-5p	PRNCR1 acts as a sponge RNA, binding to specific microRNAs (miRNAs) and modulating the expression of genes<br>related to cell proliferation, migration and tumor invasion.	162<br>190	Arman et al., (2023)	doi.org/10.1016/j.gene.2023.147232
99	FGD5-AS1 - Uninfected<br>FGD5-AS1 - Infected<br>(ENSG00000225733)	miR-153-3p / MCL1, miR-195-5p / NUAK2<br>miR-497-5p / MACC1, miR-577 / LRP6 e β-catenina	FGD5-AS1 interacts with components of the JNK/c-Jun and NF-κB signaling pathways, influencing the expression of genes<br>related to inflammation and immune response.	1,083<br>1,139	Arman et al., (2023)	doi.org/10.1016/j.gene.2023.147232
100	ZEB2-AS1 - Uninfected<br>ZEB2-AS1 - Infected<br>(ENSG00000238057)	miR-122-5p, miR-142-3p, ZEB2	ZEB2-AS1 regulates the expression of the ZEB2 gene, a transcription factor essential for the development and<br>function of immune cells, such as $CD8^+$ T lymphocytes and myeloid cells.	0<br>0	Arman et al., (2023)	doi.org/10.1016/j.gene.2023.147232
