# Supplementary material for: SARS-CoV-2 reshapes m6A methylation in long noncoding RNAs of human lung cells
Source: NAR Mol Med. 2025 Sep 30;2(4):ugaf034. doi: 10.1093/narmme/ugaf034 (PMC12628319; doi:10.1093/narmme/ugaf034)
Supplement: ugaf034_Supplemental_Files [file ugaf034_Supplemental_Files.zip › Supplementary Table S2.pdf]

Supplementary Table S2. LncRNAs involved in inflammation regulation selected from the curated set of 100 lncRNAs in uninfected cells (U) and infected cells (I).

	lncRNA	Reads	Target	Function	Reference	
1	MALAT1 - U	18	NF-κB	Induced TNF-α, IL-6, IL-1 and CCL2	Enguita et al. (2022), Dai et al.(2018), Rodrigues et ., (2021)	doi: 10.7150/thno.73268, doi.org/10.1080/03008207.2018.1439480, doi:10.1111/omi.12351
	MALAT1 - I	19			Tian et al., (2018), Hao et al., (2020) and Xu et. (2024)	doi 10.1038/s41419-018-0925-y, doi.org/10.1016/j.virusres.2020.197907, doi.org/10.1016/j.ncrna.2024.01.015
2	NEAT1 - U	3,655	NF-κB	Induced TNF-α, IL-6, and IL-1, NLRP3, as well as CCl2, Ccl3, and CXCL10	Dai et al., (2018); Moazzam-Jazi et al., (2021), Vishnubalaji et al., (2020)	doi.org/10.1080/03008207.2018.1439480, doi: 10.1111/jcmm.16596, doi:10.3390/genes11070760
	NEAT1 - I	4,532			Tian et al., (2018); Meydan et al., (2020), Rodrigues et ., (2021)	doi 10.1038/s41419-018-0925-y; doi: 10.3389/fimmu.2020.590870, doi:10.1111/omi.12351
3	KCNQ1OT1 - U	7,949	NF-κB	Role in cell survival and regulation of gene expression and	Dai et al., (2018; Tian et al., (2018)	doi.org/10.1080/03008207.2018.1439480, doi 10.1038/s41419-018-0925-y
	KCNQ1OT1 - I	9,440		Interact with upregulated TLR2		
4	RAD51-AS1 - U	54	NF-κB	Induced IL-6, TNF-α, CCL2	Morenikeji et al.(2021)	doi: 10.3389/fbioe.2020.582953
	RAD51-AS1 - I	41				
5	NORAD - U	3,587	NF-κB	Induced IL-6, IL-10, CSF3, CXCL10, TNF-α	Morenikeji et al. (2021)	doi: 10.3389/fbioe.2020.582953
	NORAD - I	4,467			Yang et al., (2021)	doi.org/10.3389/fimmu.2021.700184
6	PIRAT - U	9,867	NF-κB	Reduce alarmin production	Aznaourova et al.(2022)	doi.org/10.1073/pnas.2120680119
	PIRAT - I	11,686				
7	LUCAT1 - U	8,521	NF-κB	Induced expression of proinflammatory markers	Aznaourova et al. (2022)	doi.org/10.1073/pnas.2120680119
	LUCAT1 - I	9,912		CXCL2 and CXCL8		
8	TUG1 - U	35	NF-κB	Regulates inflammation by sponging this miRNA, which	Tayel et al., (2022)	doi: 10.7150/ijbs.72318 ,
	TUG1 - I	47		may influence the activation of factors such as NF-κB	Wang et al. (2022)	doi.org/10.1186/s42494-022-00080-6
9	(ANRIL) CDKN2B-AS1 - U	14,154	NF-κB	NF-κB activation promotes the expression of pro-inflammatory genes.	Xiaoting et al., (2019), Hu et al., (2019)	10.1186/s40246-019-0240-4, doi.org/10.1016/j.biochi.2018.10.011
	(ANRIL) CDKN2B-AS1 - I	16,471		inflammasome responses	Li et al., (2019), Badr et al., (2023)	10.18632/aging.101863, 10.1186/s12879-023-08564-7
10	GAS5 - U	2,247	TNF-α/IL-10	Suppresses NF-κB activation, reducing the release of pro-inflammatory	Ayeldeen et al., (2024), Curci et al., (2024)	doi 10.2174/0929867330666230515144133, doi 10.3389/fphar.2024.1448136
	GAS5 - I	2,655		cytokines such as TNF-α, IL-6, and IL-1β.	Li et al., (2017), Xiao and Wang (2023)	doi 10.1007/s10620-017-4831-4, 10.2174/1381612829666230517102205
11	LINC00305 - U	10,722	NF-κB	LINC00305 enhances inflammation by activating the NF-κB signaling pathway in	Morenikeji et al.(2021)	doi: 10.3389/fbioe.2020.582953
	LINC00305 - I	12,563		monocytes, contributing to the upregulation of pro-inflammatory genes	Bergara-Muguruza et al. (2023)	doi: 10.20517/jtgg.2023.14
12	LINC00473 - U	7,681	NF-κB	Regulation of pro-inflammatory cytokines; interaction with signaling pathways.	Saha et al., (2021)	doi.org/10.3390/ncrna7040074
	LINC00473 - I	9,170	P65, STAT3, CREB		Laha et al., (2021)	doi.org/10.1016/j.heliyon.2021.e06395
13	LINC00273 - U	16	NF-κB	LINC00273 may modulate the immune response by promoting the secretion of	Chattopadhyay et al., (2022)	10.3389/fimmu.2022.1035111
	LINC00273 - I	38		pro-inflammatory cytokines, IL-6 and TNF-α, activation of NF-κB signaling pathways		
14	LINC02574 -U	1,104	NF-κB	Acts by inhibiting viral replication through the positive regulation of the	Zhang et al., (2023)	doi.org/10.3390/ijms24087248
	LINC02574 -I	1,073		innate immune response.		
15	CASC2 - U	12,819	NF-κB, MAPK/ERK, PI3K/AKT	Regulates the expression of cytokines such as IL-6, TNF-α, and IL-1β;	Ayoub et al., (2024)	doi.org/10.1186/s40246-024-00578-9
	CASC2 - I	15,102		Regulation of inflammatory signaling pathways.		
16	ROR1-AS1 - U	5,991	NF-κB	ROR1-AS1 is implicated in the activation of the NF-κB signaling pathway, a central	Chattopadhyay et al. (2022)	10.3389/fimmu.2022.1035111
	ROR1-AS1 - I	7,058		regulator of inflammatory responses		
17	BISPR - U	13,594	JAK-STAT, NF-κB	Regulation of BST2 (Tetherin); positive regulator of BST2 and part of the	Enguita et al., (2022)	doi: 10.7150/thno.73268
	BISPR - I	15,728		interferon-stimulated innate immune response		
18	MIR155HG - U	1,348	NF-Kb	miR-155 Precursor, Regulation of Innate and Adaptive Immune Responses	Rai et al., (2022)	doi: 10.1128/mbio.02510-22
	MIR155HG - I	1,804		Amplification of Inflammatory Signaling	Enguita et al., (2022)	doi: 10.7150/thno.73268
19	USP30 antisense RNA 1 - U	2	USP30	Indirectly, USP30-AS1 influence inflammatory responses by regulating mitochondria	Enguita et al., (2022)	doi: 10.7150/thno.73268
	USP30 antisense RNA 1 - I	5		which is central to the production of reactive oxygen species (ROS)		
20	lncATV - U	2,174	TBK1 (TANK-binding kinase 1)	Inhibition of RLR (RIG-I-like receptors) Signaling	van Solingena et al., (2022)	doi.org/10.1073/pnas.2210321119
	lncATV - I	2,717	IRF3, RIG-I (DDX58), MDA5	Interference with TBK1 and IRF3	Enguita et al., (2022)	doi: 10.7150/thno.73268
21	CHROMR - U	8,287	IL-6 and TNF-α	Promote the transcription of ISGs	van Solingena et al., (2022)	doi.org/10.1073/pnas.2210321119
	CHROMR - I	9,603				
22	RDUR - U	11,922	NF-κB	Modulate RIG-I innate immune response mediated by (Retinoic Acid-Inducible Gene I)	Chen et al., (2021)	doi.org/10.3389/fimmu.2021.672165
	RDUR - I	13,965	IRF3 and IRF7	receptor; RIG-I–dependent antiviral response regulator RNA		
23	NRIR - U	4,678	CMPK2, STAT1, STAT2,	By Uninfectedling the activation of ISGs, NRIR may function as a modulator	Sefatjoo et al., (2024)	doi.org/10.1016/j.cyto.2023.156495
	NRIR - I	5,382	IRF3, IRF7	of inflammation, influencing protective and pathological responses	Lin et al., (2023)	doi:10.3389/fcell.2023.1229393
24	C1RL antisense RNA 1 - U	2,562	C1RL	Studies indicate that C1RL-AS1 is overexpressed in children with	Arman et al., (2023)	doi.org/10.1016/j.gene.2023.147232
	C1RL antisense RNA 1 - I	3,069		pneumonia caused by Influenza A virus, as well as in infected A549 cells	Turjya et al., (2020)	doi: 10.2217/fvl-2020-0188
25	IFNG-AS1 - U	6,927	NF-κB (p50/p65)	Modulates IFN-γ expression and enhances the antiviral response.	Laha et al., (2021)	doi.org/10.1016/j.heliyon.2021.e06395
	IFNG-AS1 - I	8,538				
26	THRIL - U	3.787	NF-κB	Induces TNF-α; TNF- and HNRNPL-related immunoregulatory activity;	Rahni et al., (2023)	doi.org/10.1016/j.virusres.2023.199214
	THRIL - I	4,323		amplifies innate immune response		
27	UCA1 - U	137	NF-κB	Negatively regulates CD8 T cell activation through PD-L1	Chattopadhyay et al., (2022)	doi 10.3389/fimmu.2022.1035111
	UCA1 - I	129		Positively regulates TNF-a, IL6, IL1b, Suppressed Inflammatory Response	Yang et al., (2024)	doi.org/10.1038/s41417-024-00734-2
28	MEG3 - U	6,599	NF-κB	Negatively regulates inflammation, inhibits NF-κB,	Turjya et al., (2020)	doi: 10.2217/fvl-2020-0188
	MEG3 - I	7,392		and modulates epigenetic mechanisms.		
29	DANCR - U	5,902	NF-κB	Regulation of cell differentiation; promotion of cell proliferation and survival.	Laha et al., (2021)	doi.org/10.3390/ncrna7040074
	DANCR - I	7,421	IL-6, TNF-α		Meydan et al., (2020)	doi: 10.3389/fimmu.2020.590870
30	HAND2-AS1 - U	12,643	NF-κB	Regulation of IL-6	Arman et al., (2023)	doi.org/10.1016/j.gene.2023.147232
	HAND2-AS1 - I	14,692				
31	LINC00511 - U	14,194	NF-κB	Competing endogenous RNA (ceRNA), interacting miRNA, modulating target expression	Taheri et al., (2021)	doi.org/10.1186/s12879-021-06248-8
	LINC00511 - I	16,440		May modulate miR-29b-3p and miR-150, regulate the inflammatory response		
32	SNHG6 - U	2,189	NF-κB	Regulating the cytokine storm.	Taheri et al., (2021)	doi.org/10.1186/s12879-021-06248-8
	SNHG6 - I	2.381		Decreased SNHG6 expression in COVID-19 suggests modulating inflammatory response		
33	LINC00278 - U	9,734	NF-κB	Potential biomarker during SARS-CoV-2 infection.	Cheng et al., (2021)	doi: 10.1111/jcmm.16444
	LINC00278 - I	11,510			Talotta et al., (2021)	doi.org/10.1016/j.bbadis.2021.166291
34	LINC01619 - U	14,926	NF-κB	LINC01619 has been identified as a potential biomarker during SARS-CoV-2 infection	Cheng et al., (2021)	doi: 10.1111/jcmm.16444
	LINC01619 - I	17,445		due to its altered expression profile and possible involvement in immune regulation	Zhong et al.,(2022)	doi 10.3389/fmolb.2022.975322
