# Supplementary material for: SARS-CoV-2 reshapes m6A methylation in long noncoding RNAs of human lung cells
Source: NAR Mol Med. 2025 Sep 30;2(4):ugaf034. doi: 10.1093/narmme/ugaf034 (PMC12628319; doi:10.1093/narmme/ugaf034)
Supplement: ugaf034_Supplemental_Files [file ugaf034_Supplemental_Files.zip › Supplementary Table S3.pdf]

Supplementary Table S3. UCA1 transcript ENST00000397381.4 m6A sites.

Infected					Uninfected				
Position	# reads	p-modified	kmer	mod-ratio	Position	# reads	p-modified	kmer	mod-ratio
1814	36	0.0047	TAACC	0.0000	1814	54	0.0027	TAACC	0.0000
1806	43	0.0386	TAACT	0.0000	1806	54	0.0363	TAACT	0.0000
1787	35	0.0438	AAACA	0.0000	1787	44	0.0455	AAACA	0.0227
1771	42	0.0029	AAACC	0.0000	1771	53	0.0165	AAACC	0.0000
1712	37	0.1896	TGACC	0.0541	1712	46	0.4483	TGACC	0.2391
1703	36	0.0004	TAACC	0.0000	1703	46	0.0007	TAACC	0.0000
1662	29	0.3498	AGACA	0.2414	1662	40	0.2056	AGACA	0.0500
1608	33	0.7137	GAACT	0.4545	1608	41	0.8781	GAACT	0.4146
1508	24	0.7577	AGACT	0.4167	1508	38	0.5626	AGACT	0.3421
1499	24	0.2085	AAACT	0.0417	1499	38	0.0502	AAACT	0.0000
1492	25	0.0223	AGACC	0.0000	1492	33	0.1280	AGACC	0.0606
1455	24	0.1782	TAACT	0.0417	1455	36	0.3736	TAACT	0.2222
1414	21	0.5217	GAACT	0.1905	1414	26	0.5185	GAACT	0.1923
1362	73	0.5067	AAACT	0.1644	1362	72	0.4323	AAACT	0.1667
1337	77	0.0374	AAACA	0.0130	1337	75	0.0282	AAACA	0.0000
1315	63	0.8943	GGACA	0.3968	1315	63	0.7628	GGACA	0.2857
1232	67	0.1630	AGACC	0.0597	1232	70	0.0697	AGACC	0.0143
1218	72	0.7968	GAACT	0.3472	1218	77	0.9069	GAACT	0.5065
1210	76	0.6999	GAACT	0.3158	1210	78	0.6255	GAACT	0.3333
1160	77	0.2007	AGACA	0.0649	1160	73	0.2024	AGACA	0.0822
1122	70	0.9274	GGACA	0.6143	1122	65	0.9237	GGACA	0.7231
1083	75	0.8043	GGACA	0.3600	1083	65	0.7781	GGACA	0.3385
1070	73	0.1588	AGACC	0.0411	1070	71	0.1979	AGACC	0.0845
1023	70	0.8731	GGACA	0.5429	1023	67	0.7633	GGACA	0.4030
929	81	0.0143	AAACA	0.0000	929	75	0.0472	AAACA	0.0267
896	81	0.5860	AGACC	0.3086	896	78	0.7037	AGACC	0.3718
863	63	0.5220	AGACC	0.2063	863	67	0.4019	AGACC	0.1045
847	70	0.9601	GGACC	0.5286	847	65	0.9449	GGACC	0.4923
730	80	0.0900	AAACA	0.0125	730	77	0.0387	AAACA	0.0130
726	73	0.3285	AGACA	0.1096	726	73	0.4542	AGACA	0.1096
663	53	0.9189	AGACA	0.6792	663	55	0.9203	AGACA	0.7455
615	74	0.4353	GGACC	0.1622	615	71	0.4400	GGACC	0.1831
568	68	0.1196	AGACC	0.0441	568	63	0.1009	AGACC	0.0317
563	53	0.8646	GGACC	0.3585	563	44	0.5869	GGACC	0.1818
545	73	0.0946	AAACT	0.0548	545	67	0.1778	AAACT	0.0448
536	64	0.0504	TGACC	0.0000	536	65	0.1524	TGACC	0.1077
498	68	0.0397	AGACA	0.0000	498	63	0.0343	AGACA	0.0000
464	76	0.0537	TAACT	0.0132	464	74	0.0553	TAACT	0.0000
437	73	0.2538	GGACC	0.1233	437	68	0.0772	GGACC	0.0294
402	71	0.0641	AGACT	0.0282	402	68	0.0353	AGACT	0.0000
355	72	0.5255	GAACA	0.3194	355	72	0.4750	GAACA	0.3194
342	41	0.2427	AGACC	0.1707	342	48	0.2383	AGACC	0.1458
280	62	0.2191	AGACC	0.0968	280	60	0.2355	AGACC	0.1333
267	68	0.1016	GGACT	0.0294	267	68	0.0792	GGACT	0.0147
237	59	0.0732	TGACA	0.0169	237	64	0.0595	TGACA	0.0156
228	63	0.2264	GGACA	0.0794	228	57	0.2468	GGACA	0.0702
180	60	0.0183	TAACA	0.0000	180	60	0.0147	TAACA	0.0000
151	40	0.2048	AGACT	0.0750	151	42	0.2551	AGACT	0.1667
