# Supplementary material for: SARS-CoV-2 reshapes m6A methylation in long noncoding RNAs of human lung cells
Source: NAR Mol Med. 2025 Sep 30;2(4):ugaf034. doi: 10.1093/narmme/ugaf034 (PMC12628319; doi:10.1093/narmme/ugaf034)
Supplement: ugaf034_Supplemental_Files [file ugaf034_Supplemental_Files.zip › Supplementary Table S4.pdf]

Supplementary Table S4. NORAD transcript ENST00000565493.1 m6A sites.

Infected					Uninfected				
**Position**	**# reads**	**p-modified**	**kmer**	**mod_ratio**	**Position**	**# reads**	**p-modified**	**kmer**	**mod_ratio**
2175	21	0.0279	AAACT	0.0000	2059	20	0.3372	GGACA	0.1000
2192	21	0.6368	AGACT	0.3810	2120	21	0.0235	TGACA	0.0000
2215	21	0.3359	TGACC	0.1905	2175	20	0.0629	AAACT	0.0500
2334	26	0.0011	TAACC	0.0000	2192	22	0.5352	AGACT	0.2273
2364	25	0.0122	TAACA	0.0000	2215	20	0.3198	TGACC	0.1000
2385	23	0.2985	GGACA	0.0870	2334	23	0.0068	TAACC	0.0000
2459	29	0.0193	TAACA	0.0000	2364	22	0.0090	TAACA	0.0000
2483	30	0.5022	AAACT	0.3333	2385	26	0.2104	GGACA	0.1154
2520	30	0.0581	GAACC	0.0000	2459	30	0.0295	TAACA	0.0000
2626	32	0.0235	TAACT	0.0000	2483	27	0.8923	AAACT	0.4444
2661	23	0.6390	GGACA	0.1304	2520	30	0.0950	GAACC	0.0333
2723	25	0.2127	TGACA	0.0800	2626	33	0.0437	TAACT	0.0000
2742	23	0.0479	AAACT	0.0000	2661	25	0.0914	GGACA	0.0800
2746	25	0.0335	TAACA	0.0000	2723	32	0.0150	TGACA	0.0000
2799	25	0.8391	GGACT	0.4800	2742	32	0.0831	AAACT	0.0000
2817	23	0.0090	TAACA	0.0000	2746	35	0.0264	TAACA	0.0000
2833	25	0.5221	TGACA	0.3600	2799	30	0.6675	GGACT	0.3000
2895	23	0.1302	AGACC	0.0435	2817	32	0.0120	TAACA	0.0000
2918	29	0.0954	AGACA	0.0345	2833	31	0.3710	TGACA	0.1935
2933	31	0.0094	TAACC	0.0000	2895	26	0.0784	AGACC	0.0385
2983	22	0.1220	GGACA	0.0455	2918	31	0.0441	AGACA	0.0000
2987	28	0.0757	AAACT	0.0357	2933	35	0.0031	TAACC	0.0000
3006	33	0.0384	AAACA	0.0303	2983	28	0.0413	GGACA	0.0000
3069	37	0.0455	TAACA	0.0000	2987	29	0.0202	AAACT	0.0000
3234	36	0.1558	TGACC	0.0556	3006	35	0.0230	AAACA	0.0000
3296	30	0.1415	GGACA	0.0667	3069	37	0.0324	TAACA	0.0000
3350	38	0.2173	TGACA	0.0526	3234	40	0.1610	TGACC	0.1000
3369	40	0.0350	AAACT	0.0000	3296	33	0.0558	GGACA	0.0303
3412	41	0.0970	AGACT	0.0732	3350	38	0.0286	TGACA	0.0000
3423	41	0.0075	AAACA	0.0000	3369	40	0.0481	AAACT	0.0250
3441	41	0.0036	AAACC	0.0000	3412	38	0.0720	AGACT	0.0526
3538	39	0.1241	AGACA	0.0513	3423	37	0.0315	AAACA	0.0000
3607	44	0.0147	AAACT	0.0000	3441	34	0.0060	AAACC	0.0000
3625	48	0.0268	AAACA	0.0000	3538	44	0.0856	AGACA	0.0227
3742	568	0.2004	GGACC	0.0599	3607	47	0.0173	AAACT	0.0000
3765	461	0.0069	AAACC	0.0000	3625	50	0.0156	AAACA	0.0000
3862	584	0.4933	GAACT	0.1884	3742	433	0.2539	GGACC	0.0901
3967	48	0.0071	TAACA	0.0000	3765	502	0.0089	AAACC	0.0000
3986	47	0.0166	TAACA	0.0000	3862	494	0.4703	GAACT	0.2024
4054	45	0.0228	AAACT	0.0000	3967	52	0.0065	TAACA	0.0000
4079	44	0.0854	AGACT	0.0227	3986	52	0.0075	TAACA	0.0000
4178	42	0.0012	TAACC	0.0000	4054	51	0.1018	AAACT	0.0392
4327	40	0.1407	TGACC	0.0750	4079	48	0.2305	AGACT	0.1042
4356	38	0.0159	AGACA	0.0000	4178	51	0.0005	TAACC	0.0000
4463	45	0.0102	TAACC	0.0000	4327	51	0.2030	TGACC	0.1176
4513	39	0.1041	GGACA	0.0513	4356	37	0.0327	AGACA	0.0000
4557	43	0.1724	TGACA	0.0698	4463	51	0.0016	TAACC	0.0000
4573	42	0.0159	AAACT	0.0000	4513	38	0.2003	GGACA	0.0789
