# Supplementary material for: SARS-CoV-2 reshapes m6A methylation in long noncoding RNAs of human lung cells
Source: NAR Mol Med. 2025 Sep 30;2(4):ugaf034. doi: 10.1093/narmme/ugaf034 (PMC12628319; doi:10.1093/narmme/ugaf034)
Supplement: ugaf034_Supplemental_Files [file ugaf034_Supplemental_Files.zip › Supplementary Table S5.pdf]

Supplementary Table S5. GAS5 transcript ENST00000430245.1 m6A sites.

Infected					Uninfected				
**Position**	**# reads**	**p-modified**	**kmer**	**mod-ratio**	**Position**	**# reads**	**p-modified**	**kmer**	**mod_ratio**
207	119	0.0598	GGACA	0.0168	207	74	0.1290	GGACA	0.0270
266	137	0.1470	TAACT	0.0511	266	82	0.0627	TAACT	0.0122
294	123	0.0407	AGACA	0.0081	294	81	0.0402	AGACA	0.0247
337	124	0.1500	GGACC	0.0484	337	81	0.2219	GGACC	0.0494
383	123	0.3937	TGACT	0.1382	383	76	0.3772	TGACT	0.1447
411	119	0.2127	TGACT	0.1092	411	81	0.3492	TGACT	0.1852
448	132	0.5013	GGACT	0.2121	448	89	0.5396	GGACT	0.2022
481	146	0.0277	AGACC	0.0000	481	98	0.0234	AGACC	0.0000
495	148	0.1117	AAACT	0.0338	495	98	0.0746	AAACT	0.0306
511	157	0.1337	TGACC	0.0318	511	103	0.1743	TGACC	0.0680
605	152	0.1580	AAACT	0.0461	605	105	0.0746	AAACT	0.0190
650	146	0.1015	AGACT	0.0479	650	100	0.0679	AGACT	0.0200
687	140	0.0300	AAACA	0.0000	687	87	0.0349	AAACA	0.0000
