# Supplementary material for: SARS-CoV-2 reshapes m6A methylation in long noncoding RNAs of human lung cells
Source: NAR Mol Med. 2025 Sep 30;2(4):ugaf034. doi: 10.1093/narmme/ugaf034 (PMC12628319; doi:10.1093/narmme/ugaf034)
Supplement: ugaf034_Supplemental_Files [file ugaf034_Supplemental_Files.zip › Supplementary Table S6.pdf]

Supplementary Table S6. NEAT1 transcript ENST00000501122.2 m6A sites.

Infected					Uninfected				
**Position**	**# reads**	**p-modified**	**kmer**	**mod-ratio**	**Position**	**# reads**	**p-modified**	**kmer**	**mod-ratio**
16648	35	0.5182	AGACT	0.2571	16648	37	0.7732	AGACT	0.4054
16713	92	0.1457	AAACT	0.0870	16713	73	0.1155	AAACT	0.0548
16809	124	0.2156	GAACC	0.0968	16809	117	0.2971	GAACC	0.1368
17846	623	0.1163	AGACC	0.0385	17846	570	0.1058	AGACC	0.0368
17869	561	0.0148	AAACC	0.0036	17869	542	0.0116	AAACC	0.0018
17966	583	0.2934	GAACC	0.1475	17966	501	0.2754	GAACC	0.1397
17997	265	0.0498	AGACC	0.0189	17997	270	0.0339	AGACC	0.0074
18025	447	0.2768	TGACA	0.1320	18025	418	0.3066	TGACA	0.1435
