# Supplementary material for: SARS-CoV-2 reshapes m6A methylation in long noncoding RNAs of human lung cells
Source: NAR Mol Med. 2025 Sep 30;2(4):ugaf034. doi: 10.1093/narmme/ugaf034 (PMC12628319; doi:10.1093/narmme/ugaf034)
Supplement: ugaf034_Supplemental_Files [file ugaf034_Supplemental_Files.zip › Supplementary Table S7.pdf]

Supplementary Table S7. Comparison of lncRNA expression in Calu-3 cells and COVID-19 patients from transcriptome data PRJNA718349 samples GSE171110 (44 COVID-19 patients and 10 controls) and GSE157103 (100 COVID-19 patients and 26 controls).

Current study - 940,040 reads	lncRNA	Mapped Reads	Normalized	Fold Change
Uninfected Calu-3	BISPR	13,594	0.014461	
Uninfected Calu-3	GAS5	2,247	0.002390	
Uninfected Calu-3	NORAD	3,587	0.003816	
Uninfected Calu-3	UCA1	137	0.000146	
Uninfected Calu-3	NEAT1	3,665	0.003899	
**Current study - 1,055,956 reads**				
Infected Calu-3	BISPR	15,728	0.014895	1.03
Infected Calu-3	GAS5	2,655	0.002514	1.05
Infected Calu-3	NORAD	4,467	0.004230	1.11
Infected Calu-3	UCA1	129	0.000122	0.84
Infected Calu-3	NEAT1	4,532	0.004292	1.10
**GSE171110 - 399,305,389 reads**				
Healthy donor	BISPR	4,280,049	0.010719	
Healthy donor	GAS5	985,552	0.002468	
Healthy donor	NORAD	1,797,669	0.004502	
Healthy donor	UCA1	10,609	0.000027	
Healthy donor	NEAT1	6,267,369	0.015696	
**GSE171110 - 1,954,976,820 reads**				
COVID-19_Severe	BISPR	22,959,970	0.011744	1.10
COVID-19_Severe	GAS5	5,238,627	0.002680	1.09
COVID-19_Severe	NORAD	9,271,695	0.004743	1.05
COVID-19_Severe	UCA1	84,915	0.000043	1.63
COVID-19_Severe	NEAT1	35,892,347	0.018359	1.17
**GSE157103 - 1,910,042,028 reads**				
Control	BISPR	19,970,748	0.010456	
Control	GAS5	6,066,688	0.003176	
Control	NORAD	6,323,553	0.003311	
Control	UCA1	31,318	0.000016	
Control	NEAT1	32,759,530	0.017151	
**GSE157103 - 6,546,086,478 reads**				
COVID-19	BISPR	74,178,636	0.011332	1.08
COVID-19	GAS5	21,907,663	0.003347	1.05
COVID-19	NORAD	23,438,948	0.003581	1.08
COVID-19	UCA1	132,912	0.000020	1.24
COVID-19	NEAT1	127,836,019	0.019529	1.14
