# Supplementary material for: SARS-CoV-2 reshapes m6A methylation in long noncoding RNAs of human lung cells
Source: NAR Mol Med. 2025 Sep 30;2(4):ugaf034. doi: 10.1093/narmme/ugaf034 (PMC12628319; doi:10.1093/narmme/ugaf034)
Supplement: ugaf034_Supplemental_Files [file ugaf034_Supplemental_Files.zip › Supplementary Table S8.pdf]

Supplementary Table S8. Expression of genes encoding IFN and IFN receptors in Calu-3 cells and COVID-19 patients from transcriptome data PRJNA718349 samples GSE171110 (44 COVID-19 patients and 10 controls) and GSE157103 (100 COVID-19 patients and 26 controls).

Current study - 940,040 reads	Genes	Mapped Reads	Normalized	Fold Change
Uninfected Calu-3	IFN-α (IFNA1) (ENSG00000197919)	0	0.000001	
Uninfected Calu-3	IFN-α (IFNA2) (ENSG00000188379)	0	0.000001	
Uninfected Calu-3	IFN-β (ENSG00000171855)	0	0.000001	
Uninfected Calu-3	IFNAR1 (ENSG00000142166)	14,996	0.015953	
Uninfected Calu-3	IFNAR2 (ENSG00000159110)	12,646	0.013453	
Uninfected Calu-3	IFN-γ (ENSG00000111537)	0	0.000001	
Uninfected Calu-3	IFNGR1 (ENSG00000027697)	4,013	0.004269	
Uninfected Calu-3	IFNGR2 (ENSG00000159128)	16,603	0.017662	
**Current study - 1,055,956 reads**				
Infected Calu-3	IFN-α (IFNA1) (ENSG00000197919)	0	0.000001	1.00
Infected Calu-3	IFN-α (IFNA2) (ENSG00000188379)	0	0.000001	1.00
Infected Calu-3	IFN-β (ENSG00000171855)	77	0.000073	68.55
Infected Calu-3	IFNAR1 (ENSG00000142166)	17,393	0.016471	1.03
Infected Calu-3	IFNAR2 (ENSG00000159110)	14,815	0.014030	1.04
Infected Calu-3	IFN-γ (ENSG00000111537)	0	0.000001	1.00
Infected Calu-3	IFNGR1 (ENSG00000027697)	4,679	0.004431	1.04
Infected Calu-3	IFNGR2 (ENSG00000159128)	19,201	0.018184	1.03
**GSE171110 - 399,305,389 reads**				
Healthy donor	IFN-α (IFNA1) (ENSG00000197919)	10	0.00000003	
Healthy donor	IFN-α (IFNA2) (ENSG00000188379)	10	0.00000003	
Healthy donor	IFN-β (ENSG00000171855)	2	0.00000001	
Healthy donor	IFNAR1 (ENSG00000142166)	4,715,117	0.01180830	
Healthy donor	IFNAR2 (ENSG00000159110)	4,461,176	0.01117234	
Healthy donor	IFN-γ (ENSG00000111537)	763	0.00000191	
Healthy donor	IFNGR1 (ENSG00000027697)	2,783,689	0.00697133	
Healthy donor	IFNGR2 (ENSG00000159128)	4,861,158	0.01217404	
**GSE171110 -1,954,976,820**				
COVID-19_Severe	IFN-α (IFNA1) (ENSG00000197919)	10	0.00000001	0.20
COVID-19_Severe	IFN-α (IFNA2) (ENSG00000188379)	17	0.00000001	0.35
COVID-19_Severe	IFN-β (ENSG00000171855)	19	0.00000001	1.94
COVID-19_Severe	IFNAR1 (ENSG00000142166)	25,350,349	0.01296708	1.10
COVID-19_Severe	IFNAR2 (ENSG00000159110)	23,932,575	0.01224187	1.10
COVID-19_Severe	IFN-γ (ENSG00000111537)	2,268	0.00000116	0.61
COVID-19_Severe	IFNGR1 (ENSG00000027697)	14,954,418	0.00764941	1.10
COVID-19_Severe	IFNGR2 (ENSG00000159128)	26,277,208	0.01344119	1.10
**GSE157103 - 1,910,042,028 reads**				
Control	IFN-α (IFNA1) (ENSG00000197919)	9	0.000000005	
Control	IFN-α (IFNA2) (ENSG00000188379)	30	0.000000016	

Control	IFN-β (ENSG00000171855)	35	0.000000018	
Control	IFNAR1 (ENSG00000142166)	22,549,153	0.011805579	
Control	IFNAR2 (ENSG00000159110)	21,257,754	0.011129469	
Control	IFN-γ (ENSG00000111537)	54,430	0.000028497	
Control	IFNGR1 (ENSG00000027697)	10,135,814	0.005306592	
Control	IFNGR2 (ENSG00000159128)	24,680,304	0.012921341	
**GSE157103 - 6,546,086,478 reads**				
COVID-19	IFN-α (IFNA1) (ENSG00000197919)	35	0.000000005	1.13
COVID-19	IFN-α (IFNA2) (ENSG00000188379)	114	0.000000017	1.11
COVID-19	IFN-β (ENSG00000171855)	143	0.000000022	1.19
COVID-19	IFNAR1 (ENSG00000142166)	84,140,029	0.012853486	1.09
COVID-19	IFNAR2 (ENSG00000159110)	78,885,847	0.012050841	1.08
COVID-19	IFN-γ (ENSG00000111537)	233,608	0.000035687	1.25
COVID-19	IFNGR1 (ENSG00000027697)	37,271,278	0.005693673	1.07
COVID-19	IFNGR2 (ENSG00000159128)	92,158,143	0.014078357	1.09
